# Impact of gut microbiota on endometriosis: linking physical injury to mental health

**DOI:** 10.3389/fcimb.2025.1526063

**Published:** 2025-07-07

**Authors:** Li Yuanyue, Hu Qian, Liu Ling, Yang Liufeng, Ge Jing, Wu Xiaomei

**Affiliations:** ^1^ Department of Gynecology, The First People’s Hospital of Yunnan Province, Kunming, China; ^2^ The Affiliated Hospital of Kunming University of Science and Technology, Kunming, Yunnan, China; ^3^ Yunnan Provincial Key Laboratory of Clinical Virology, Kunming, China; ^4^ Department of Information, The First People’s Hospital of Yunnan Province, Kunming, China

**Keywords:** endometriosis, gut microbiota, immune dysfunction, estrobolome, complement system

## Abstract

Endometriosis is widely recognized as a chronic, inflammatory, and heterogeneous condition that can manifest in various anatomical locations within females. It is marked by estrogen-driven cycles of bleeding, cell proliferation, and fibrosis involving ectopic endometrial glands and stroma cells located outside the uterus. The limited understanding of its etiology and complex pathogenesis has created obstacles in achieving early diagnosis and developing effective treatments with minimal side effects. Consequently, endometriosis requires more in-depth research to unravel its pathogenesis. The gut microbiota, a key player in chronic diseases, significantly influences bodily metabolism and immune regulation. Emerging evidence links the gut microbiota to inflammation, estrogen metabolism, and immune responses—key factors in the onset and progression of endometriosis. This review examines the various mechanisms through which endometriosis and the gut microbiota interact, aiming to inspire new strategies for preventing and early treating endometriosis.

## 1 Introduction

Endometriosis is a chronic systematic disorder characterized by dysmenorrhea, persistent pelvic pain, infertility, and pelvic masses, predominantly affecting women of reproductive age (Saunders and Horne, 2021). Estimated to impact 5–10% of women who desire children, this condition currently has no known cure (Taylor et al., 2021; Saunders et al., 2024). Diagnosis typically requires surgical confirmation of endometrial-like tissue (“lesions”) located outside the uterus, a process that can take an average of seven years from the onset of symptoms (Horne and Missmer, 2022). Women with endometriosis frequently experience fatigue, depression, and other mental health issues before and after diagnosis (Cuffaro et al., 2024; Sinai et al., 2024). Overall, endometriosis significantly affects both physical health and mental well-being. The complex and multi-faceted nature of endometriosis, emphasizes not only the physical but also the psychological burden of the disorder. The prolonged diagnostic delay, averaging seven years, underscores the challenges in early identification, likely due to the disease’s complex pathology and overlapping symptoms with other conditions. The mental health toll on affected women—manifesting in fatigue, depression, and other psychological issues—indicates that endometriosis is not solely a gynecological condition but a systemic issue impacting the quality of life.

The gut microbiota, a relatively new area of scientific research, is estimated to contain around 10¹³ bacteria, a number comparable to the total count of human cells. In contrast, the bacterial gut microbiome, which refers to the collective gene pool encoded by these gut bacteria, contains roughly 100 times more unique genes than the human genome. This intricate ecosystem, shaped by a long history of co-evolution between microbes and their hosts, and its close relationship with human health, has increasingly captured the interest of researchers over the past few decades (Round and Mazmanian, 2009; Visconti et al., 2019; Van Hul et al., 2024). The fact that the gut microbiome contains far more unique genes than the human genome suggests that our microbial partners may contribute to functions beyond human genetic capabilities. This rich genetic reservoir likely supports essential roles in metabolism, immune function, and even mental health. The intricate co-evolutionary relationship hints at a deep biological interdependence and its link to health underlines the need for further exploration. Continued research into the gut microbiota may unlock new insights into disease prevention, personalized medicine, and overall well-being.

There is substantial evidence linking gut microbiota to the pathogenesis of endometriosis, spanning from physical injury to mental health effects like depression. In this process, the gut microbiota influences the immune system (Fan et al., 2023), estrobolome (Alghetaa et al., 2023), and brain-gut axis (Salliss et al., 2022). The connection between gut health and endometriosis symptoms—including both physical and mental health effects—suggests a need for a broader, systemic approach to the disease, extending beyond traditional gynecological perspectives. However, the specifics of these linkages remain underexplored. This review aims to synthesize recent advancements in our understanding of endometriosis pathobiology, with a focus on the inflammatory, metabolic, and pain pathways influenced by the gut microbiota. By examining its relationship to endometriosis, physical injury, and mental health, we provide a comprehensive overview of this emerging field. In the concluding sections, we discuss ongoing clinical trials and consider how recent insights may lead to effective non-surgical treatment options.

## 2 Endometriosis: pathophysiology and symptoms

### 2.1 Current conceptions in pathogenesis of endometriosis

The etiology of endometriosis is complex, with multiple factors contributing to the development of this disorder (Ochoa Bernal and Fazleabas, 2024). Several theories have been proposed to explain its origin, with Sampson’s retrograde menstruation theory currently the most widely accepted. In 1927, Sampson suggested that endometriosis results from the reflux of endometrial fragments through the fallopian tubes during menstruation, which then attach and implant, forming peritoneal and ovarian lesions (Sampson, 1927). However, a limitation of this theory is that it cannot account for the fact that while retrograde menstruation occurs in up to 90% of reproductive-aged women, only 6–10% go on to develop endometriosis (Ochoa Bernal and Fazleabas, 2024). Another prominent theory, Coelomic Metaplasia, explains the occurrence of endometriosis in females who do not menstruate, such as premenstrual adolescent girls, postmenopausal women, or those with total hysterectomies, as well as patients with Mayer–Rokitansky–Küster–Hauser (MRKH) syndrome, a condition associated with the absence of a uterus (Troncon et al., 2014). Another influential theory, the Embryonic Rest Theory, was introduced by Von Recklinghausen and Russell in the 1890s. It proposes that embryonic cell remnants of Müllerian origin within the peritoneal cavity may differentiate into functional endometrial tissue under certain conditions. This could explain rare cases of endometriosis in men, as Müllerian cell rests exist in males and may reside anywhere along the migration pathway of the Müllerian system.

Another proposed mechanism, the lymphatic dissemination theory, suggests that endometrial tissue spreads through the vascular and lymphatic systems, which accounts for its presence in lymph nodes and distant locations. The Tissue Injury and Repair (TIAR) Theory posits that endometriosis results from trauma, involving an estrogen-driven mechanism that is abnormally amplified in reproductive organs16. Meanwhile, Quinn’s “Denervation–Reinnervation” Theory proposes that endometriotic cells may migrate outside the uterine cavity following nerve injuries in the uterus and uterosacral ligaments, often after challenging deliveries or persistent strain during defecation (Quinn, 2011). The Stem Cell Theory also presents an intriguing perspective, proposing that stem cells from the basalis layer of the endometrium can migrate through the fallopian tubes or spread via lymphatic and vascular routes during menstruation, establishing endometriotic lesions beyond the peritoneal cavity (Cordeiro et al., 2022). Lastly, the genetic/epigenetic theory—one of the most recent explanations—suggests that genetic and epigenetic alterations, alongside overlapping cellular processes, create the conditions that contribute to endometriosis development (Koninckx et al., 2019).

### 2.2 Pathophysiology of endometriosis

Cell proliferation, invasion, and angiogenesis—characteristics common to both endometriosis and malignant tumors—are driven by chronic inflammation that promotes malignancy (Tulandi and Vercellini, 2024). Factors such as hormones, the immune microenvironment, and inflammation are crucial in the progression of endometriosis. The growth of endometriotic implants is particularly driven by estradiol, a key estrogen steroid hormone (Bulun et al., 2019; Smolarz et al., 2021). Ectopic endometrial tissues exhibit an overexpression of estrogen receptor beta (ER-beta), which in turn suppresses the activity of estrogen receptor alpha (ER-alpha). This suppression diminishes the ability of ER-alpha to induce the progesterone receptor, ultimately leading to enhanced cell survival and inflammation via ER-beta activation (Patel et al., 2017; Taylor et al., 2021). Progesterone typically inhibits estrogen-driven endometrial proliferation, induces decidualization of the endometrium, and acts as an anti-inflammatory agent. However, in endometriosis, progesterone resistance was first observed in *in-vitro* studies, where progesterone fails to stimulate the production of retinoic acid, the resulting deficiency in retinoic acid contributes to elevated estradiol levels in endometriotic lesions, thereby promoting their growth. Additionally, endometriosis is marked by a low ratio of progesterone receptor isoform B (PR-B) to progesterone receptor isoform A (PR-A) (Patel et al., 2017).

Endometriosis is a chronic inflammatory disease that is dependent on estrogen, with endocrine and immunological interactions playing a vital role in its pathogenesis. The overproduction of estrogen and resistance to progesterone lead to dysfunction in the peritoneal immune microenvironment. The increased expression of estrogen receptor alpha (ER-alpha) and estrogen receptor beta (ER-beta) enhances macrophage recruitment and M2 polarization while diminishing phagocytic activity and the production of pro-inflammatory cytokines, thereby inhibiting the inflammatory response. Estrogen also reduces the cytotoxic activity of natural killer (NK) cells due to decreased autophagy in endometrial stromal cells (ESCs), promoting immune evasion by ESCs and contributing to the development of endometrial lesions. Additionally, hormones have a significant impact on the activity of neutrophils, T cells, and B cells, as well as on the expression of pro-inflammatory cytokines. Additionally, endometriosis is considered a chronic systemic disease involving various pro-inflammatory and inflammatory components, such as microRNAs, cytokines, and stem cells (Taylor et al., 2021; Lamceva et al., 2023). Numerous links between inflammation and endometriosis have been identified (Oală et al., 2024). Despite these insights, no single theory has fully explained the pathogenesis of endometriosis. However, advancements in technology are unraveling an increasing number of complexities through multi-omics approaches, including single-cell sequencing and transcriptome analysis (Fonseca et al., 2023; Liu et al., 2023; Sarsenova et al., 2024).

### 2.3 Symptoms and complications

**Figure 1:**
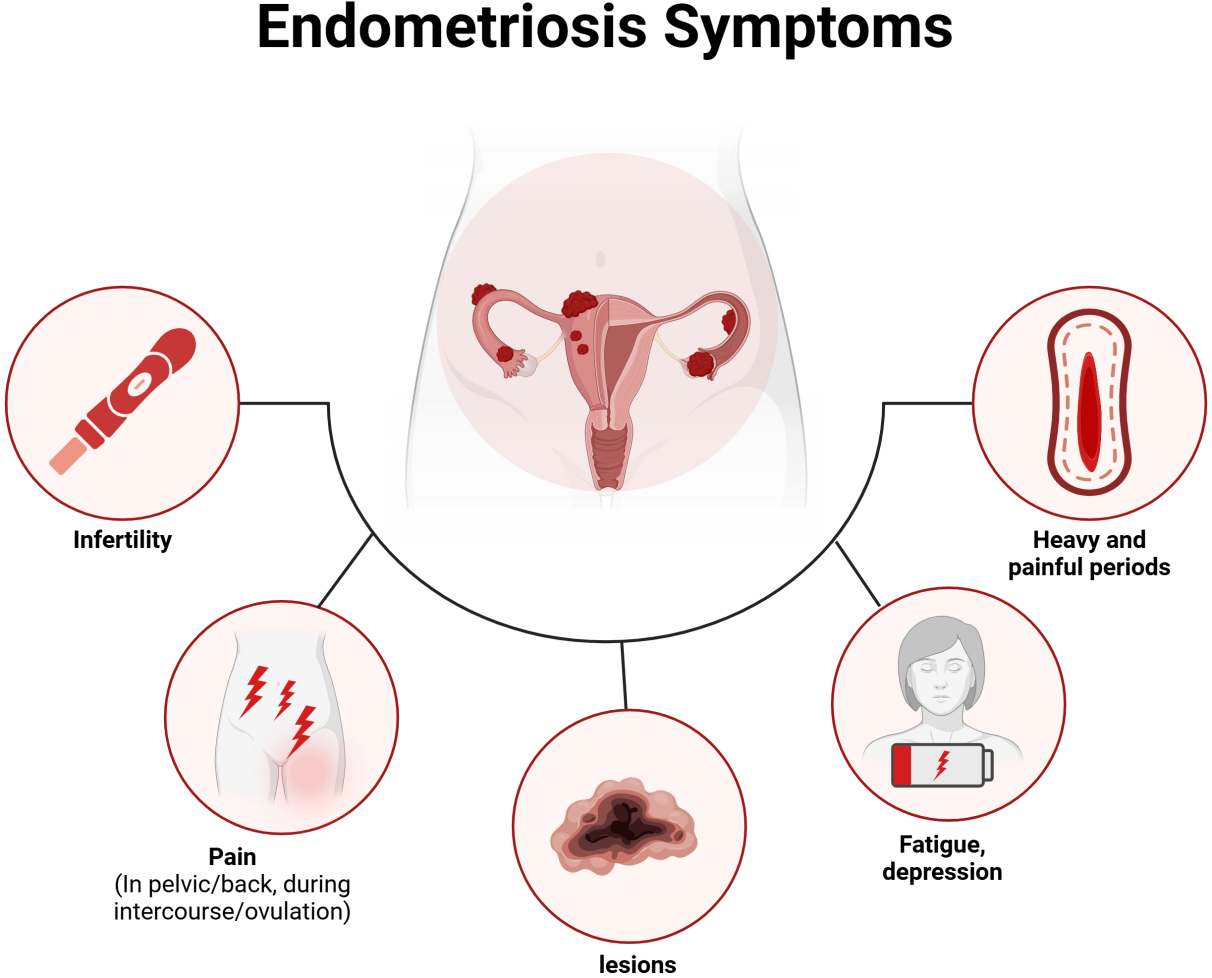
The common symptoms associated with endometriosis.

Endometriosis does not exhibit pathognomonic signs or symptoms that are unique to a disease localized in the pelvis; rather, it manifests symptoms that are often associated with a variety of both gynecological and non-gynecological conditions. This condition is associated with a wide range of symptoms, with the most prevalent being pain, bowel and bladder issues, as well as symptoms related to other chronic pain conditions, such as fatigue and depression (Bulun et al., 2019). Notably, studies indicate no direct correlation between the type or location of endometriosis and the symptoms experienced (Pant et al., 2023). The variability of symptoms—some of which may not occur in all patients—contributes to the well-documented delays in diagnosis, as they often overlap with other conditions. Many individuals with endometriosis report experiencing dysmenorrhea and chronic pelvic pain during adolescence or early adulthood (Sasamoto et al., 2022). However, these painful symptoms are frequently underestimated and dismissed as normal or transient experiences for young women (Martire et al., 2023). The impact of endometriosis extends to multiple aspects of life (Zondervan et al., 2018; Nassiri Kigloo et al., 2024; Thiel et al., 2024), including obstetrical complications, unnoticed organ dysfunction, an increased risk of ovarian cancer, strained relationships, elevated levels of depression and anxiety, financial burdens from expensive fertility treatments, and absenteeism from work. For those facing “unexplained infertility,” the absence of a diagnosis can lead to an emotionally challenging journey and a significant decline in health-related quality of life (Nezhat et al., 2024). As shown in **Figure 1**, we provide an overview of the symptoms of endometriosis, including both clinical somatic and mental symptoms.

### 2.4 Gut microbiota: composition and functions

The gut microbiota, also known as the gut microbiome (GM), refers to the diverse array of microorganisms that reside in the human gastrointestinal (GI) tract. Although it is commonly referred to as the gut microbiome, this community includes not only bacteria but also fungi, viruses, and helminths. Bacteria make up a substantial part of the gut microbiome and are more thoroughly characterized compared to the other less understood components. From the earliest stages of human life, the gut microbiome plays a vital role in various physiological processes, such as nutrient absorption, maintaining the integrity of the GI lining, regulating immune and endocrine functions, and safeguarding against pathogenic threats (Salliss et al., 2022; Fasano et al., 2024).

There is growing evidence linking alterations in the microbiome to a diverse array of diseases, including inflammatory bowel disease (IBD), liver disorders, obesity, diabetes, and even some neurological conditions (Round and Mazmanian, 2009). The idea of microbiome imbalance has been linked to various disease states (Round and Mazmanian, 2009; Wang M-Y. et al., 2024). However, while correlations can be identified, establishing causation and predicting disease progression based on microbiome composition remains a complex challenge.

Research has revealed the existence of unique bacterial communities throughout the female reproductive tract, establishing a continuum of microbiotas that extends from the vagina to the ovaries. Furthermore, a growing body of evidence underscores the significance of gut microbiota in the progression of endometriosis (Chen et al., 2017; Huang et al., 2021; Jiang et al., 2021; Li et al., 2021; Talwar et al., 2022; Alghetaa et al., 2023; Cuffaro et al., 2024; Liu et al., 2024; Wang M. et al., 2024). A more thorough understanding of the relationship between microbiota and endometriosis has unveiled their potential role in the development of this condition (van Barneveld et al., 2022; Chadchan et al., 2023; Lamceva et al., 2023). All of these factors may significantly contribute to the pathogenesis of endometriosis, particularly in light of recent advances in understanding the pathological mechanisms involved, such as adhesion, invasion, and angiogenesis.

## 3 Gut microbiota and endometriosis: mechanistic links

### 3.1 Immune and inflammatory responses

**Figure 2:**
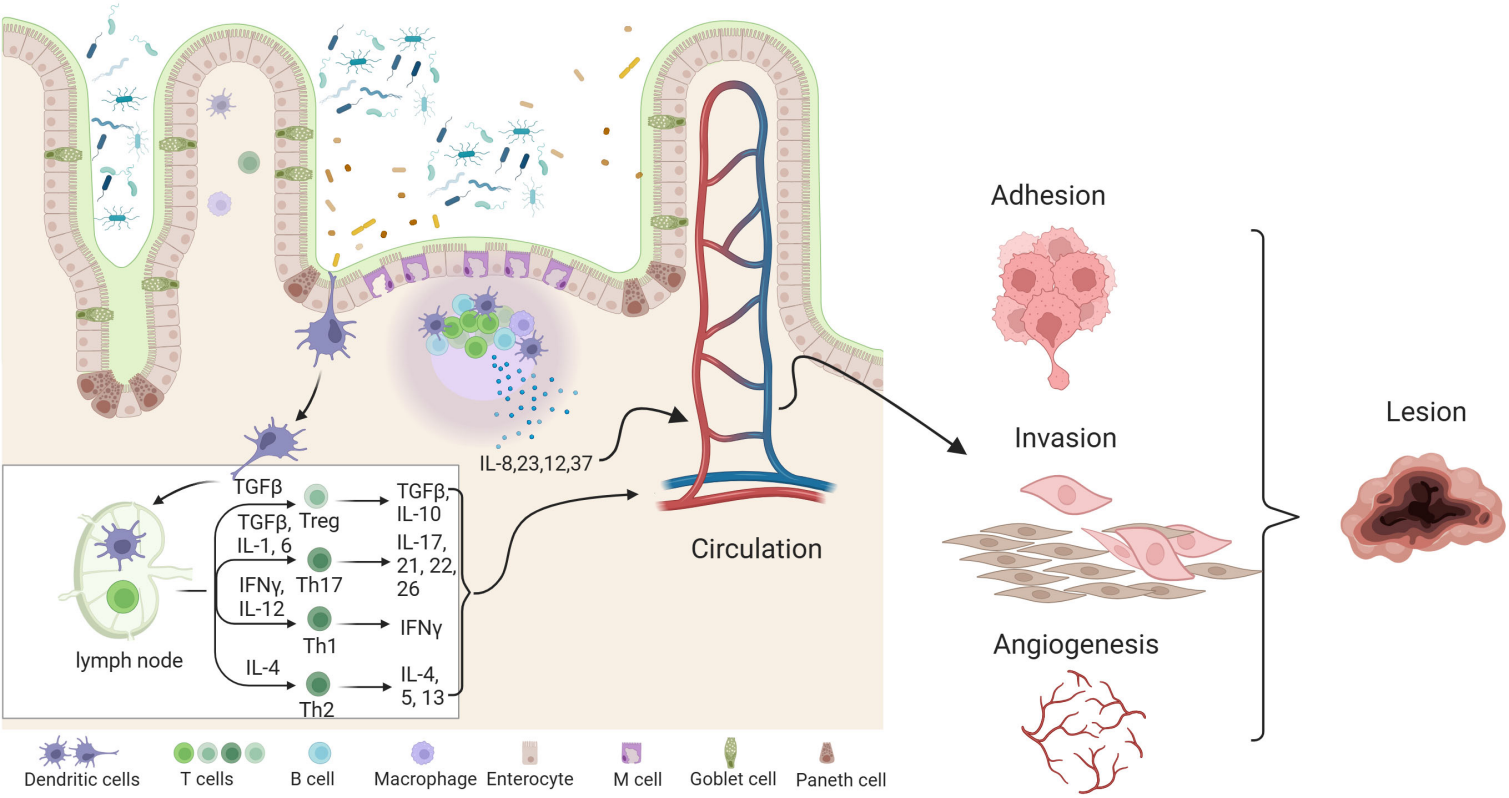
Effects of gut dysbiosis and its metabolite pathophysiology of endometriosis. combined effects of gut dysbiosis and altered microbial metabolites contribute to the systemic inflammatory environment and hormonal disturbances, exacerbating endometriosis symptoms and promoting lesion growth.

Imbalances in the gut and female reproductive tract microbiomes disrupt normal immune function, prompting inflammatory responses that elevate pro-inflammatory cytokines, impair immune surveillance, and alter immune cell profiles (Visconti et al., 2019). This immune imbalance can lead to chronic inflammation, fostering conditions that support increased adhesion and angiogenesis, potentially driving the cycle of endometriosis onset and progression. As shown in **Figure 2**, studies have indicated that the inflammatory response in the peritoneal fluid of EMs patients is active, with an enhanced aggregation of inflammatory cells and a significant difference in the expression of various inflammatory factors compared to healthy women (Samimi et al., 2019). In healthy women without endometriosis, immune cells, including macrophages and natural killer (NK) cells, are recruited and activated to remove endometrial debris that has refluxed into the peritoneal cavity (Reis et al., 2024). Furthermore, chemokines released by immune cells create a pro-inflammatory environment that prevents the implantation of ectopic endometrial tissue. In contrast, patients with endometriosis (EMs) have a unique immune-inflammatory microenvironment, where ectopic endometrial cells in menstrual blood can evade immune surveillance and develop into endometriosis lesions (Suryawanshi et al., 2014).The occurrence and development of endometriosis involve a variety of immune cells, including lymphocytes, dendritic cells, and macrophages. These immune components play a crucial role in driving the implantation and growth of endometriosis (Vallvé-Juanico et al., 2019; Chen et al., 2023). Studies have shown that ectopic endometrial cells exhibit distinct immunophenotypes and biological activities, which can activate neutrophils, macrophages, natural killer cells, and dendritic cells in the abdominal cavity, thereby contributing to immune-related inflammatory responses (Vetvicka et al., 2016).

Lymphocytes are the smallest white blood cells, produced by lymphoid organs, and play essential roles in immune functions. They are classified into T lymphocytes (T cells), B lymphocytes (B cells), and natural killer (NK) cells. Retrospective studies have confirmed that endometriosis (EMS) is associated with alterations in T cell activity (de Barros et al., 2017). Regulatory T cells participate in the angiogenesis of EMS (Wang et al., 2017). The development and growth of eEMS lesions are linked to the activation of both systemic and local humoral responses, which are driven by an increase in Th2 lymphocyte numbers (Antsiferova et al., 2005). Gogacz et al. (2016)found that the percentage ratio of Th17 cells in ascites was positively correlated with the severity of EMS, suggesting that a decrease in the ratio of Th17 to total CD4+ T lymphocyte subsets may contribute to excessive proliferation of ectopic endometrial tissue, thereby driving disease progression. In the pathogenesis of EMS, B lymphocytes may also play a role by secreting autoantibodies (Osuga et al., 2011). Riccio et al. (2018) observed that reduced cytotoxicity of NK cells can promote ectopic endometrial adhesion and proliferation, leading to the formation of lesions, which helps to explain the immune escape mechanism of endometrial cells. Furthermore, the proportion of uterine natural killer (uNK) cells was found to be significantly lower in ectopic lesions. Drury et al. (2018) suggest that the reduction in uNK cells associated with ectopic endometrial cells may play a role in the early formation of these lesions.

A dendritic cell (DC) is an antigen-presenting cell that can recognize and capture antigens, promoting the differentiation of naive T cells and thereby inducing an antigen-specific immune response. In the lesion, activated DCs stimulate T cells, contributing to the damage of early EMs lesions (Stanic et al., 2014). The expansion of myeloid-derived suppressor cells (Mo-MDSCs) and regulatory T cells (Tregs) derived from monocytes in EMs contributes to the creation of an intraperitoneal immunosuppressive microenvironment in EMs patients, thereby promoting disease progression. Compared to healthy women, patients with EMs exhibit higher concentrations of Tregs, which suppress the body’s immune response to ectopic lesions. This inhibition fosters an anti-inflammatory environment, facilitating the ectopic implantation and growth of endometrial cells.

The reduced phagocytic activity of macrophages may contribute to the pathogenesis of EMS, although the exact mechanism remains unclear (Liu et al., 2019). Since EMs lesions involve recurrent tissue damage and repair, M1 macrophages may play a role in mediating tissue damage and triggering an inflammatory response during the early stages of endometriosis. Subsequently, M2 macrophages contribute to tissue repair and support the growth of endometriosis lesions (Duan et al., 2018). The expansion of myeloid-derived suppressor cells (Mo-MDSCs) and regulatory T cells (Tregs) derived from monocytes in EMs contributes to the development of an intraperitoneal immunosuppressive microenvironment, thereby promoting the progression of the disease (Chen et al., 2018). Thus, it seems that the mononuclear phagocyte system plays a role in promoting the growth of aberrant blood vessels in EMs, with its production of proinflammatory cytokines contributing to the establishment of an inflammatory environment that accelerates disease progression.

In cases of endometriosis, however, elevated levels of cytokines in the peritoneal fluid, such as interleukin (IL)-6, IL-1β, IL-8, tumor necrosis factor (TNF)-α, and transforming growth factor (TGF)-β, contribute to the development of a chronically inflamed peritoneal environment (Chen et al., 2023; Nati et al., 2024). Both the innate and adaptive immune systems contribute to the development of ectopic lesions in endometriosis. Dendritic cells promote angiogenesis and lesion formation, while neutrophils are recruited early to release vascular endothelial growth factor (VEGF) and neutrophil extracellular traps (NETs). This shift in the immune cell profile creates a chronic inflammatory environment that fosters neuro-angiogenesis and immune evasion, thereby exacerbating lesion progression (Fan et al., 2023). Both pro-inflammatory and anti-inflammatory molecules appear to play a role in the development of endometriosis. Emerging evidence suggests that platelets and regulatory T cells (Tregs) may promote type 2 immunity within the lesional microenvironment, facilitating lesion growth and fibrogenesis. This is achieved through increased recruitment of macrophages, M2 macrophages, Tregs, Th2, and Th17 cells, along with reduced Th1 cell activity. Additionally, the release of cytokines such as IL-37, IL-23, IL-17, and others further drives lesion progression (Podgaec et al., 2007; Sisnett et al., 2024; Gogacz et al., 2016; Xiao et al., 2020; Shi et al., 2022; Li et al., 2024). Neutrophils produce pro-inflammatory cytokines, including vascular endothelial growth factor (VEGF), IL-8, IL-12, CXCL10, and CXCL12, all of which can contribute to the progression of the disease (Munrós et al., 2019). Neutrophils can activate key mediators by secreting proteases, such as neutrophil elastase, which play a role in promoting the initial development of EMs (Takamura et al., 2016). In summary, neutrophils secrete biochemical factors that facilitate the growth, invasion, and angiogenesis of endometriotic cells. Research has shown that Th17 cells, which co-secrete interleukin (IL)-10 and IL-17A, promote the growth, adhesion, invasion, and deep infiltration of endometrioid cells (ESCs), thereby accelerating the progression of EMs (Chang et al., 2017). IL-8 is implicated in all stages of EMs development. It can induce the adhesion, invasion, implantation, and proliferation of ectopic endometrial cells, while also potentially protecting these cells from apoptosis, thereby aiding their survival and persistence (Sikora et al., 2017). IL-33 can initiate both local and systemic signaling, stimulating the proliferation of EMs lesions and inducing angiogenesis. *In vitro* stimulation of endometrial epithelial cells, endothelial cells, and EMS epithelial cells with IL-33 promotes the production of pro-inflammatory and angiogenic molecules. Biomolecules involved in or related to immune responses are collectively referred to as immune molecules. Their primary biological function is to bind to specific factors, facilitating immune cell recognition, intercellular signaling, and interactions between cells and tissues.

The complement system constitutes a crucial component of the human body’s first line of defense against microbial pathogens, while also playing essential roles in immune surveillance, infection control, and the regulation of inflammation. In the context of endometriosis, increasing attention has been directed toward understanding the involvement of the complement system. Notably, as early as 1988, a study reported decreased levels of complement components C3 and C4 during the follicular phase of the menstrual cycle in patients with endometriosis, highlighting a potential link between complement activity and disease pathogenesis (Meek et al., 1988). Subsequently, in 2007, another study reported elevated levels of complement components C3c, C4, and SC5b-9 in the serum compared to the peritoneal fluid of women with endometriosis. Interestingly, the levels of iC3b were found to be higher in the peritoneal fluid relative to the serum. Furthermore, the study demonstrated that the concentrations of C3c, C4, and SC5b-9 in both the peritoneal fluid and serum were significantly higher in women with endometriosis compared to healthy controls. In contrast, the levels of iC3b in both compartments — peritoneal fluid and serum — were significantly lower in patients with endometriosis than in the control group (Kabut et al., 2007). In patients with adenomyosis, treatment with danazol for eight weeks was associated with an increase in C4 levels, while C3 levels showed a decrease (Ota et al., 1992). Additionally, researchers observed significantly elevated levels of C1q, mannose-binding lectin (MBL), and C1 inhibitor (C1INH) in the peritoneal fluid (PF) of women with endometriosis compared to healthy controls (p < 0.0001), with these differences being particularly pronounced during the early stages of the disease (Sikora et al., 2018). Through co-expression analysis and experimental validation, researchers demonstrated that the upregulation of complement components (C1S, C1QA, C1R, and C3) was positively correlated with the expression of tissue factor (TF) in endometriotic (EM) tissues (Yu et al., 2021). The C5a serum levels were higher in patients with EM than in controls but not associated with the severity or clinical findings (Rahal et al., 2023). Using immunohistochemistry (IHC), researchers confirmed that complement factor 7 (C7) was highly expressed in both endometriosis and ovarian cancer tissues, whereas normal endometrial tissues exhibited little to no mRNA expression. Moreover, the protein expression levels of C7 were consistent with the corresponding gene expression data (Suryawanshi et al., 2014). The serum levels of adipsin and complement factor- H(CFH) were found to be significantly increased in women with endometriosis. What is more, a strong and positive correlation was also observed between peritoneal fluid levels of adipsin and CFH (Eşkin Tanrıverdi et al., 2025). High Mannose-binding lectin (MBL) level, a carbohydrate pattern recognition molecule—the first described recognition subcomponent of the complement lectin pathway was also found to be related to the disease severity (Toffoli et al., 2025). Currently, research on the role of the complement system in endometriosis remains fragmented and incomplete, with limited studies exploring its detailed molecular mechanisms. Among the various components of the complement system, complement component 3 (C3) has been the most extensively studied in the context of endometriosis (Kabut et al., 2007; Karadadas et al., 2020; Agostinis et al., 2021). Various studies have demonstrated that the complement system represents one of the most critical immune mechanisms involved in the clearance of endometrial debris and the regulation of the inflammatory response of ectopic endometrial tissue within the peritoneal cavity. It plays a pivotal role in the initiation and progression of endometriosis. Therefore, further in-depth investigations focusing on the complement system may offer valuable insights and provide a theoretical basis for exploring it as a potential therapeutic target for the treatment of endometriosis.

In summary, dysbiosis in the gut and female reproductive tract disrupts immune function, triggering inflammatory responses that elevate pro-inflammatory cytokines, compromise immunosurveillance, and alter immune cell profiles (Gogacz et al., 2016; Riccio et al., 2019; Suen et al., 2019; Xiao et al., 2020; Shi et al., 2022). This imbalance can cause structural and functional damage across multiple organs, potentially affecting entire bodily systems. The resulting immune dysregulation often leads to chronic inflammation, fostering conditions conducive to increased adhesion and angiogenesis, which in turn perpetuate the cycle of endometriosis onset and progression (Vallvé-Juanico et al., 2019).

### 3.2 Gut microbiota in endometriosis

Comprising trillions of microorganisms in the intestines, the gut microbiota is essential for regulating immune responses, managing inflammation, and preserving overall health (Visconti et al., 2019; Van Hul et al., 2024). Increasing evidence indicates that dysbiosis—an imbalance in gut microbiota—can contribute to various conditions beyond the gastrointestinal tract, including reproductive disorders like endometriosis.

Most of the literature on microbiota and endometriosis available so far focuses on the flora of the reproductive tract. For example, a research team from Nagoya University in Japan published a paper in Science Translational Medicine titled “*Fusobacterium* infection promotes the development of endometriosis through the phenotypic transition of endometrial fibroblasts” (Muraoka et al., 2023). This study identifies *Fusobacterium* infection as a potential causative agent of endometriosis. The researchers found elevated levels of *Fusobacterium* in the endometrium of women with endometriosis, particularly *Fusobacterium nucleatum*, which was significantly more abundant in the tissues of endometriosis (EMs) patients compared to the control group. *F. nucleatum* induces an innate immune response via its membrane lipopolysaccharide and increases the number of CD163-positive M2 macrophages, the predominant immune cells in endometriotic lesions. These M2 macrophages produce TGF-β1, and there was a marked increase in macrophages and TGF-β1 infiltrating the endometrial tissue compared to the control group. *In vitro*, assays demonstrated that even heat-inactivated *F. nucleatum* promoted M2 macrophage formation and stimulated TGF-β1 production. These findings suggest that *F. nucleatum* in the endometrium may influence the abundance of TAGLN in fibroblasts by upregulating TGF-β1 signaling. An increasing number of studies have shown that the gut microbiota also plays a critical role in endometriosis. The interaction between the immune system and gut microbiota is fundamental to maintaining immune homeostasis, influencing both local and systemic immune responses that can impact the development and progression of endometriosis. As a result, many researchers have conducted in-depth studies to explore the relationship between endometriosis and the intestinal microbiota, aiming to understand how microbial imbalances in the gut may contribute to the onset and progression of the disease. Yuan et al. (2018) used a mouse model of endometriosis to observe changes in the intestinal microbiota over time. While clear adhesion formation and typical ectopic foci were observed in the abdominal cavity 14 and 28 days after modeling, there were no significant differences in the diversity and abundance of the intestinal microbiota during these early stages. It was not until 42 days after modeling that a significant difference in β-diversity was observed between the endometriosis model group and the control group. Specifically, the intestinal microbiota in the endometriosis model group was enriched in *Firmicutes*, whereas the control group was enriched in *Bacteroidetes*. This finding suggests that the disease has a cumulative effect on the intestinal microbiota over time and that effective intervention during this process may help prevent the further progression of pathological changes. Cao et al. (2020) also found that the *Firmicutes*/*Bacteroidetes* ratio increased in rats after the successful establishment of the endometriosis model, indicating that endometriosis causes an imbalance in the intestinal microbiota. This result was further confirmed in a study of rhesus monkeys (Bailey and Coe, 2002). Moreover, in the monkeys with endometriosis, the number of *Lactobacillus* decreased in older individuals, while the numbers of Gram-negative aerobic bacteria and facultative anaerobic bacteria increased (Bailey and Coe, 2002).

However, animals and humans are not directly comparable. Ata et al. (2019) showed that, although the overall composition of the intestinal microbiota in women with stage 3/4 endometriosis was similar to that of healthy women, there were differences at the genus level. Women with endometriosis were more likely to have *Shigella*/*Escherichia coli* as the dominant bacteria in their fecal microbiota. This suggests that endometriosis-induced changes in gut microbiota have been observed in both animal and human studies, providing valuable data for understanding the pathological changes in the later stages of the disease. Furthermore, studies have confirmed that an imbalance in intestinal flora promotes the development of endometriosis. Chadchan et al. (2019) found that the content of *Bacteroides* in the feces of endometriosis mice was higher compared to non-endometriosis mice. After treatment with metronidazole, *Bacteroides* was no longer detectable in the feces of endometriosis mice. *Bacteroides* is known to be a gram-negative, non-sporulating anaerobe that is part of the endogenous microbiota in humans and other mammals. When treated with metronidazole, the mice exhibited smaller endometriosis lesions. It is speculated that metronidazole targets *Bacteroides* species, leading to a reduction in macrophages in the lesion, a decrease in the number of epithelial cells positive for Ki-67 (a proliferation marker), and lower concentrations of tumor necrosis factor (TNF)-α, interleukin (IL)-6, and transforming growth factor (TGF)-β1 in the peritoneal fluid. This suggests that metronidazole plays a role in inhibiting the proliferation of endometriosis lesions. A key finding of this study was that when mice were treated with metronidazole and the volume of endometriosis lesions significantly reduced, the volume of lesions in recipient mice significantly increased after receiving fecal microbiota from endometriosis mice. This indicates that the microbiota plays a crucial role in the progression of endometriosis. Due to ethical limitations, no studies have directly investigated whether human microbiota can cause disease. While the advent of 16S rRNA gene sequencing technology has enabled researchers to analyze the fecal microbiota of patients with endometriosis and identify more precise differences between patients and non-patients, it has not yet been possible to establish causality between the gut microbiota and the disease. The exact mechanism of action remains unclear. However, many researchers favor the hypothesis of bidirectional regulation between endometriosis and the gut microbiota, suggesting a complex interplay between the two.

Gut health influences the endometrium primarily through the modulation of systemic and local inflammation. The gut acts as a vital site for immune system interaction, and disturbances in gut microbiota can result in chronic low-grade inflammation. This inflammatory state can extend beyond the gut and impact distant tissues, including the endometrium. In cases of endometriosis, gut dysbiosis may worsen the inflammatory environment in the pelvic region, promoting the growth of endometrial-like tissue outside the uterus. This inflammation disrupts the normal functioning of the endometrium, impairing its roles in the menstrual cycle and fertility.

Another important way gut microbiota may affect endometrial health is through hormone regulation (Baker et al., 2017). The gut is involved in the metabolism of estrogen via the estrobolome, which consists of gut bacteria that modulate circulating estrogen levels. An imbalance in these bacteria can lead to elevated or altered estrogen levels, a key hormone in the development and progression of endometriosis. Estrogen promotes the proliferation of endometrial tissue, and disruptions in gut microbial activity can contribute to hormonal dysregulation, exacerbating endometriosis symptoms and negatively affecting reproductive health.

Moreover, gut health is essential for maintaining the integrity of the gut barrier and, by extension, the pelvic barrier, which helps prevent harmful bacterial by-products such as lipopolysaccharides (LPS) from entering the bloodstream (Su et al., 2024). When these endotoxins penetrate the barrier, they can exacerbate the inflammatory processes associated with the pathogenesis of endometriosis, negatively impacting both the endometrium and reproductive health. This underscores the vital importance of maintaining a healthy gut microbiota to support not only proper gastrointestinal function but also overall reproductive health.

**Figure 3:**
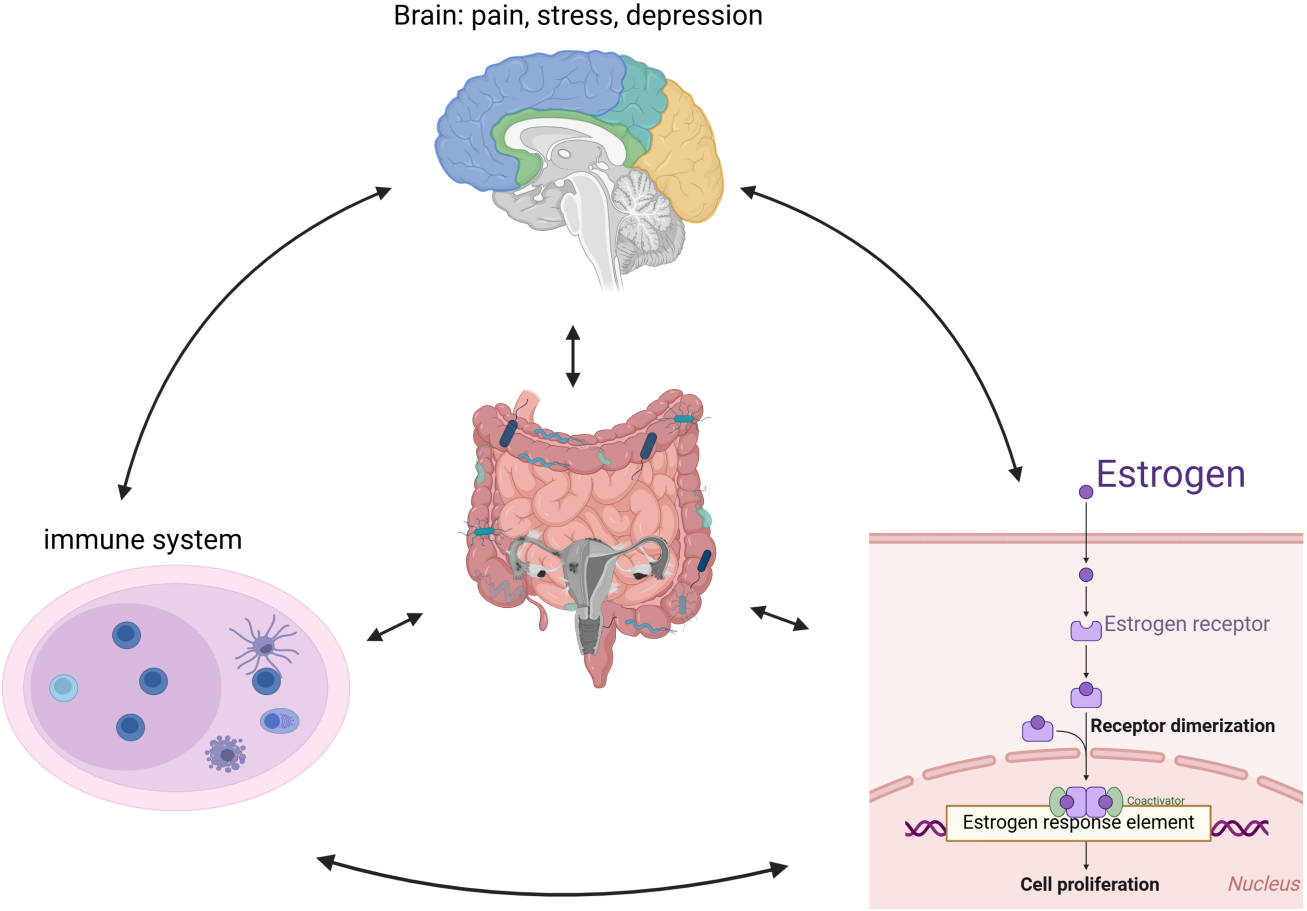
The proposed pathophysiological mechanisms underlying endometriosis development and progression.

As shown in **Figure 3**, intestinal flora is closely linked to endometriosis. Current studies suggest a strong relationship between immunity and hormone metabolism, with the gut microbiota potentially playing a significant role in the onset and progression of endometriosis. This influence may occur through various mechanisms, including mediating inflammatory responses, regulating immune function, and interfering with estrogen metabolism. Additionally, the gut microbiota may impact patients with endometriosis via the brain-gut axis, contributing to mental health challenges such as pain and depression.

In summary, existing studies on the correlation between gut microbiota and endometriosis highlight the involvement of abnormal bacteria, including Fusobacterium and others, as shown in **Table 1**. The abnormal increase in Gram-negative bacteria may influence the immune microenvironment of ectopic lesions through lipopolysaccharides (LPS), thereby contributing to disease progression and the manifestation of related symptoms. Research has shown that the LPS content in the peritoneal fluid of endometriosis patients is significantly higher compared to the control group (Kulkoyluoglu-Cotul et al., 2019). In addition, LPS can promote lesion development in endometriosis model mice (Ni et al., 2020). TLR4, a class I transmembrane protein, plays a pivotal role in the innate immune response. As an innate immune receptor, TLR4 can be recognized by the lipopolysaccharide (LPS) receptor on the surface of gram-negative bacteria. When LPS interacts with TLR4, it initiates an inflammatory cascade, releasing a large number of inflammatory mediators and causing digestive tract damage. Studies have identified the LPS-TLR4 signaling pathway in intestinal flora as a key pathogenic factor in pathogenic bacteria (or mutants), with intestinal microecological imbalance leading to increased TLR4 expression in peripheral blood monocytes and higher levels of peripheral inflammatory factors. This imbalance also causes a reduction in tight junctions between intestinal epithelial cells, increasing intestinal mucosal permeability. Additionally, intestinal microorganisms transport LPS into the bloodstream, where it binds to lipopolysaccharide-binding protein (LBP) and activates its receptor, CD14. CD14 helps LPS recognize and activate TLR4, triggering the MyD88/NF-κB pathway, which promotes the release of IL-1, IL-6, and TNF-α, thus initiating systemic inflammatory cascades that contribute to the occurrence and progression of endometriosis. In conclusion, the gut microbiota plays a significant role in the progression of endometriosis by modulating immune-related inflammatory responses. However, due to the complexity and diversity of the intestinal flora, and the intricate effects of its metabolites, the precise mechanisms underlying the role of gut microbiota in the development of endometriosis remain unclear and warrant further investigation.

**Table 1:** Gut microbiota in endometriosis including humans and animals.

Study	Location	Sample resources	Sample sizes		Diagnosis methods		Results
			Trail	Control	culturing	Sequencing	
Bailey and Coe, 2002	USA	rhesus monkeys	8	10	incubating the agar plates		Endometriosis was associated with lower Lactobacilli concentrations and higher Gram-negative bacteria concentrations. Moreover, there was a higher prevalence of intestinal inflammation in monkeys with endometriosis in comparison to healthy controls.
Yuan et al., 2018	China	Mice	22	20		16S rRNA	The *Firmicutes/Bacteroidetes* ratio was elevated in mice with endometriosis, *Bifidobacterium* was also increased in mice with endometriosis.
Chadchan et al., 2019	USA	Mice	15	14		16S rRNA	Mice with endometriosis had a higher abundance of Bacteroidetes and lower abundance of Firmicutes in their guts than mice without endometriosis.
Hantschel et al., 2019	Germany	Mice	8	8		16S rRNA	No significant effect of endometriosis induction on the composition of the bacterial microbiota was detected with experimental setup.
Ata et al., 2019	Turkey	human	14	14		16S rRNA	stool microbiome predominantly composed of Shigella/Escherichia in 2 women in the stage 3/4 endometriosis group.
Cao et al., 2020	China	Mice	8	8		16S rRNA	In endometriotic rats, the Firmicutes/Bacteroidetes ratio increased, and the abundance of Ruminococcaceae was reduced.
Ni et al., 2020	China	Mice	6	6		16S rRNA	At the phylum level, the decreased abundance of *Bacteroides* and *Firmicutes* and ratio of *Firmicutes/Bacteroides* (2.25 vs. 2.01) and the increased abundance of *Proteobacteria* and *Verrucomicrobia* (p < 0.05) are observed in the EM group. Among the top 20 abundant species at the genus level, the abundance of *Allobaculum, Akkermansia, Parasutterella*, and *Rikenella* in the EM group has increased significantly (p < 0.05), whereas the abundance of eight species of bacteria, such as *Lachnospiraceae_NK4A136_group, Lactobacillus, Bacteroides*, has decreased significantly (p < 0.05).
Shan et al., 2021	China	human	12	12		16S rRNA	The EM group exhibited reduced α diversity in their gut microbiota along with an elevated *Firmicutes/Bacteroidetes* ratio. Significant differences were observed in the abundances of various taxonomic groups, including *Actinobacteria, Tenericutes, Blautia, Bifidobacterium, Dorea*, and *Streptococcus*, between the two groups.
Svensson et al., 2021	Sweden	human	66	198		16S rRNA	Controls exhibited higher levels of both alpha and beta diversities compared to patients. At a false discovery rate of q<0.05, the abundances of 12 bacterial species, belonging to the classes *Bacilli, Bacteroidia, Clostridia, Coriobacteriia*, and *Gamma proteobacteria*, were found to differ significantly between the patient and control groups.
Le et al., 2021	USA	human	20	9		16S rRNA	GI bacterial communities were comparable between P-EOSIS and CON subjects who were not taking OCPs, but they differed significantly when OCPs were used.
Huang et al., 2021	China	human	21	20		16S rRNA	The fecal microbiota differs significantly between the control group and the EM group. Additionally, the composition of the fecal microbiota varies between patients with early and advanced stages of EM. Furthermore, the depletion of *L. Ruminococcus* in the gut may serve as a potential biomarker for endometriosis.
Le et al (Le et al., 2022). (2021)	USA	Female olive baboons	8	8		16S rRNA	Disease induction resulted in decreased levels of *Succinivibrio, Prevotella, Megasphaera, Lactobaccillus* and *CF231* at 3 months post-inoculation, but the levels of *Succinivibrio, Prevotella*, and *CF231* increased throughout disease progression from 6 to 9 months post inoculation.
Pai et al., 2023	Taiwan	human	37	35		16S rRNA	There were no significant differences in diversity and composition between individuals with and without Endometriosis in the gut microbiota.
Hicks et al., 2024	Australia	human	21	43		16S rRNA	Patients with moderate/severe endometriosis had higher levels of *Fusobacterium*.
Jimenez et al., 2024	USA	human	35	38		16S rRNA	CPP−Endo exhibited an increased abundance of rectal *Ruminococcus*.
Pérez-Prieto et al., 2024	Spain	human	136	864		shotgun metagenomic	No significant differences in diversity were found between women with endometriosis and those without.
Valdés-Bango et al., 2024	Spain	human	38	46		16S rRNA	Compared with controls, specific bacterial taxa were identified as either enriched *(Rhodospirillales, Ruminococcus gauvreauii group, Ruminococcaceae*, and *Actinomyces*) or depleted in both the gut and endometrial microbiota of adenomyosis patients.
Do et al., 2024	USA	human	33	15		16S rRNA	P-EOSIS exhibited microbial imbalance, marked by the presence of distinct GI/UG bacteria as well as changes in microbial richness and diversity.
Li et al., 2024	China	human	22	18		16S rRNA	*Proteobacteria* in the EMT group were significantly higher than those in the control group. The relative abundances of *Burkholderiales_592524* and *Sphingomonadales* in the EMT group were significantly higher than those in the CTL group.

### 3.3 Brain-gut axis and endometriosis-associated chronic pain

**Figure 4:**
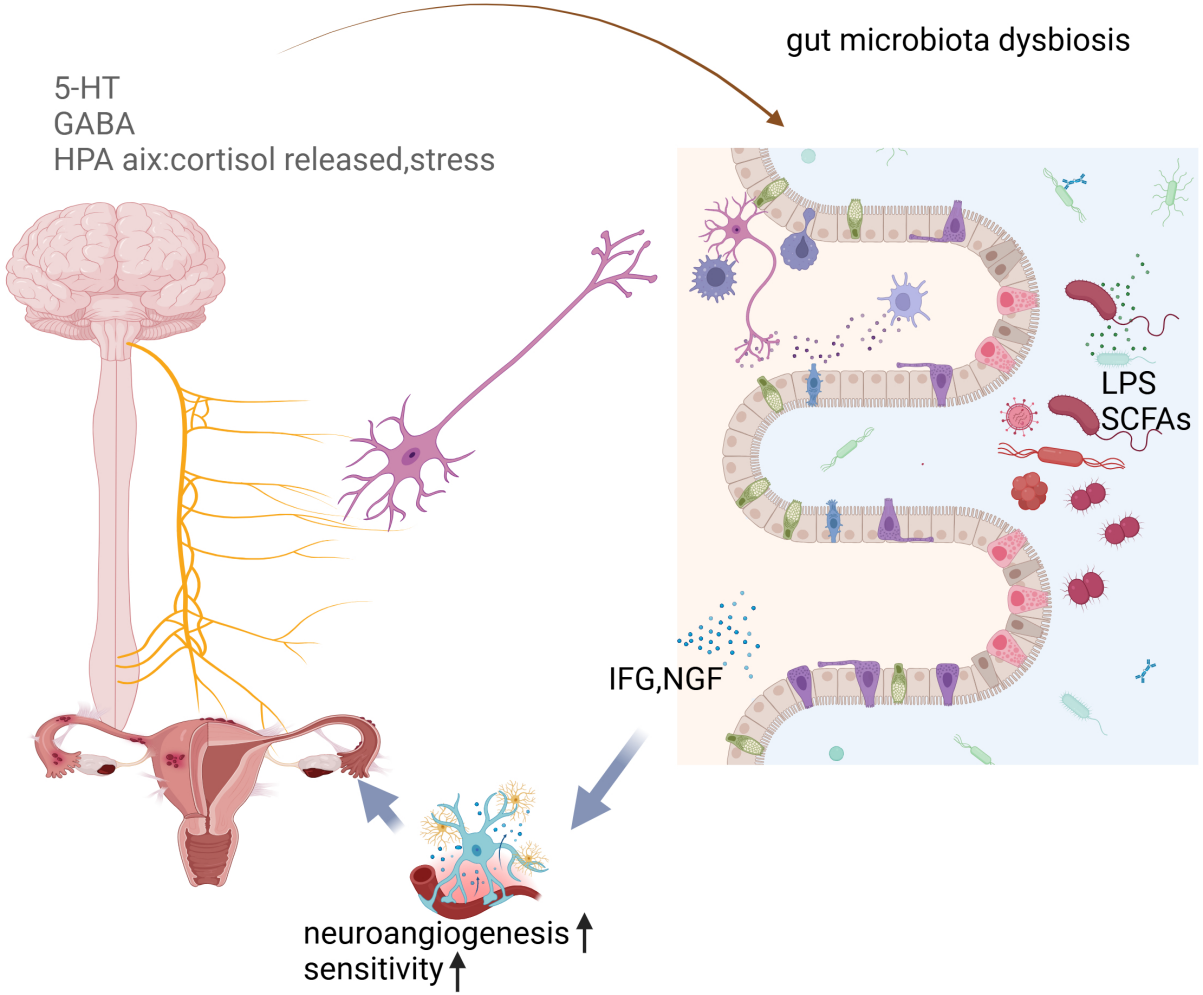
The interconnected relationship between gut microbiota imbalance, depression, and endometriosis, emphasizing how gut health impacts mental and reproductive health.

As shown in **Figure 4**, psychiatric symptoms commonly observed in women with endometriosis include fatigue, burnout, anxiety, and depression. Most of these conditions arise due to persistent pain, such as dysmenorrhea, chronic pelvic pain, and lower abdominal pain during menstruation. Pain perception occurs when biochemical signals generated by peripheral or internal nociceptive stimuli are transformed into neural signals. At the spinal cord level, these signals are either weakened or amplified before being transmitted to the cerebral cortex, where they are processed as pain (Allaire et al., 2023). Existing evidence suggests that endometriosis lesions are associated with abnormal formation of peripheral nerves and blood vessels, peripheral nerve sensitization, and morphological and functional changes in the central nervous system, all of which contribute to endometriosis-related pain. Ectopic lesions undergo repeated proliferation, swelling, and bleeding under periodic hormonal stimulation, which can activate nerve fibers in the peritoneum at the lesion site. Additionally, nerve fibers within ectopic lesions proliferate due to the elevated expression of nerve growth factor, further contributing to pain sensitivity (Maddern et al., 2020). DE pain is associated with direct pressure or irritation of the pelvic nerves. The growth and expansion of endometriotic lesions can apply pressure to surrounding tissues, including nerve fibers, leading to increased pain sensitivity and discomfort. This mechanical stimulation of the pelvic nerves is a key factor contributing to the chronic pain often experienced by women with endometriosis (Tal et al., 2019). The PF of endometriosis patients contains elevated levels of nerve growth factor (NGF), brain-derived neurotrophic factor (BDNF), and other neurotrophins such as NT-4 and NT-5. These neurotrophic factors play a significant role in neurodevelopment and are involved in the modulation of endometriosis-associated innervation, contributing to the pain experienced by patients. By promoting the growth and sensitization of nerve fibers within ectopic lesions, these factors can exacerbate the pain response in women with endometriosis (As-Sanie et al., 2016; Raffaelli et al., 2021). Nervous system sensitization can be categorized into peripheral sensitization and central sensitization. Peripheral sensitization occurs when nociceptors are activated, their threshold for stimulation is reduced, and they become more responsive to suprathreshold stimuli. This process is typically driven by inflammatory changes in the environment surrounding nerve fibers, which trigger a neuroinflammatory cascade. As a result, peripheral nerve excitability and sensitivity increase, heightening the perception of pain and leading to peripheral sensitization (Maddern et al., 2020; Raffaelli et al., 2021; Ramírez-Pavez et al., 2021). The International Association for the Study of Pain (IASP) defines central sensitization as an increased responsiveness of nociceptive neurons in the central nervous system to normal or subthreshold stimuli. Persistent inflammatory nociceptive stimuli around ectopic lesions transmit harmful signals to spinal dorsal root neurons, activating spinal microglia, which sustain pain signals. Over time, this prolonged activation leads to central sensitization, contributing to the amplification of pain perception (As-Sanie et al., 2016). The mechanism of central sensitization may help explain why chronic pelvic pain (CPP) affects approximately 30% of endometriosis patients, and why it often remains resistant to traditional surgical treatments. This sensitization process amplifies pain signals within the central nervous system, making it difficult to manage pain solely through surgical interventions.

The prolonged presence of peripheral stimuli often leads to sensitization of the central nervous system, making patients more sensitive to even mild stimuli (Nijs et al., 2014; Jensen et al., 2016; Ji et al., 2018). Endometriosis is commonly associated with dysmenorrhea, and studies show that women with dysmenorrhea exhibit different brain metabolism patterns compared to women without dysmenorrhea (Tu et al., 2013). Even when women with dysmenorrhea are not experiencing pain, their brain response to harmful stimuli is heightened (Vincent et al., 2011). Chronic pain from conditions like endometriosis can lead to structural changes in the central nervous system. For instance, in a 2012 study by Sanie et al. (As-Sanie et al., 2012), women with chronic pelvic pain (CPP) due to endometriosis showed reduced volumes in the cingulate, insula, and putamen regions, while the volume of the midbrain’s periaqueductal gray, a key area in descending pain regulation, was increased. Moreover, another study by Sanie et al. found that patients with endometriosis-related CPP had higher concentrations of excitatory neurotransmitters in the anterior insula and stronger connectivity between the anterior insula and the medial prefrontal cortex, with this increased connectivity correlating positively with the degree of pain (As-Sanie et al., 2016). Chronic pain also alters central nervous system function, affecting endocrine pathways such as the hypothalamic-pituitary-adrenal (HPA) axis. HPA axis dysfunction is often observed in chronic pain, manifesting as lower cortisol levels or a diminished stress response (Frodl and O’Keane, 2013). Over time, chronic stress may lead to elevated cortisol levels, and these levels are negatively correlated with the duration of burnout symptoms (Vincent et al., 2011).

Approximately 65% of women with endometriosis report experiencing pain symptoms (Ding et al., 2024). The gut microbiota is instrumental in regulating neurophysiological behaviors by impacting neural, endocrine, and immune pathways (Salliss et al., 2022). The relationship between the gut microbiome and the central nervous system (CNS) is bidirectional, commonly referred to as the gut microbiome-brain axis (Ustianowska et al., 2022). This axis involves immune, neural, endocrine, and metabolic pathways, enabling effective communication and interaction between various organ systems. Key players in this communication include the enteric nervous system and the sympathetic and parasympathetic divisions of the autonomic nervous system.

Growing evidence suggests that neurogenic processes contribute significantly to the development and maintenance of endometriotic lesions. Sensory, sympathetic, and parasympathetic nerves have been identified in peritoneal lesions, with a markedly higher expression compared to normal peritoneum. Furthermore, endometriotic lesions can attract their nerve supply as they invade surrounding tissues by secreting neurotrophic factors (NTFs) that promote neural sprouting within the lesions (Velho et al., 2021). This enhanced innervation may play a crucial role in hypersensitivity to touch and other stimuli, as well as persistent pain, by lowering the sensory thresholds of nociceptors. Clinical studies have indicated that patients with a higher density of nerve fibers in their endometriotic lesions report more severe pain (McKinnon et al., 2012). It is believed that these nerve fibers contribute to the development of chronic pelvic pain, with potential mechanisms linked to the gut-brain axis (Lee et al., 2023). For instance, postoperative pain relief has been associated with the influence of gut microbiota on microglial activation (Yang et al., 2022). Additionally, cytokines such as IGF and metabolites including serotonin (5-HT) and gamma-aminobutyric acid (GABA) are also involved in the gut-brain axis, further influenced by the gut microbiota (Ni et al., 2021; Kim, 2024).

### 3.4 Physical burdens following endometriosis, especially depression and anxiety

Beyond its physical effects, endometriosis (EM) profoundly impacts the emotional well-being and mental health of women, frequently leading to psychiatric symptoms, particularly anxiety and depression (Laganà et al., 2017). A study involving 7,606 women revealed that those with endometriosis were significantly more likely to report mental health challenges (P <.0001), including depression and anxiety (Gete et al., 2023). Additionally, a recent report indicated a slight increase in the risk of mental health disorders associated with endometriosis, especially in the years following diagnosis. This heightened risk emphasizes the necessity for proactive mental health screenings for individuals who are newly diagnosed with the condition (Thiel et al., 2024).

The pain related to endometriosis can trigger or worsen psychological distress, adversely affecting various dimensions of quality of life, such as physical, sexual, and social aspects (Spinoni et al., 2024). Research has shown a correlation between endometriosis and elevated levels of pro-inflammatory cytokines in the bloodstream. Furthermore, chronic stress and chronic pelvic pain (CPP) can disrupt the hypothalamic-pituitary-adrenal (HPA) axis, resulting in reduced production of inflammatory mediators within both the circulatory system and the brain, while also impacting gut microbiota composition.

Simultaneously, Shicai Xie demonstrated that the administration of LR.KY16 significantly alleviated stress-induced abnormal behaviors and physiological dysfunction (Xie et al., 2024). Additionally, gut microbiota may influence the host’s inflammation levels in the brain by regulating neurotransmitters, potentially contributing to the onset of depression. The interaction between Limosilactobacillus-3-OMDP and inflammatory markers such as IL-1β and IL-6 could represent a key pathway in the communication between the gut and the brain, with 3-OMDP emerging as a promising therapeutic target for depression (Zhong et al., 2024).

Further studies have revealed that after an imbalance in the intestinal flora, the metabolism of intestinal bacteria—such as LPS and SCFAs—can influence the central nervous system through the brain-gut axis, leading to increased production of neurotransmitters like s5HT and GABA. These changes can, in turn, affect the HPA axis. Additionally, metabolites produced by the gut microbiota, including IGF and NGF, play a role in the pain and innervation associated with endometriosis. Through these various mechanisms, the gut microbiota can contribute to psychiatric symptoms in patients with endometriosis, influencing both pain perception and emotional well-being.

### 3.5 Infertility related to endometriosis

Infertility is a significant and distressing issue for women with endometriosis, affecting both their physical and mental well-being. Studies have shown that up to 40% of women with endometriosis experience infertility, and the underlying causes are multifactorial. The role of the gut microbiota in this process is an emerging area of research, suggesting that imbalances in gut bacteria may influence the development and progression of infertility in these patients. So, what role does the gut microbiota play in this process?

Pelvic adhesion formation in endometriosis is closely associated with the activity of transforming growth factor-beta (TGF-β), a key cytokine in the inflammatory process. Research has demonstrated that endometriosis-induced dysregulation of the intestinal microbiota significantly contributes to this pathological process. In various animal models, an imbalance in the gut microbiota triggers an inflammatory response, resulting in the increased presence of peritoneal macrophages and the subsequent secretion of large amounts of TGF-β (Bailey and Coe, 2002; Yuan et al., 2018; Ata et al., 2019; Cao et al., 2020). TGF-β plays a critical role in the development of pelvic adhesions by promoting the growth of fibroblasts and influencing the production of pro-inflammatory cytokines. For instance, TGF-β1 regulates the transcription of the IL-6 gene, leading to elevated levels of IL-6 in human fibroblasts. IL-6, in turn, activates macrophages, which further stimulate the proliferation of endometrial cells. During the inflammatory process, TGF-β1 fosters adhesion between ectopic endometrial cells and stromal cells, contributing to the development of adhesions. Additionally, TGF-β1 has chemotactic effects, attracting macrophages, fibroblasts, and neutrophils to the site of injury, and promoting the secretion of extracellular matrix components such as fibronectin and collagen. These actions collectively facilitate the formation of pelvic adhesions, which are a hallmark of endometriosis (Young et al., 2017). The evidence suggests that the intestinal microbiota influences the development of pelvic adhesions via the TGF-β1 pathway. As pelvic adhesions become more severe, they interfere with the normal function of the reproductive system, leading to sperm-egg binding disorders and, ultimately, infertility in women with endometriosis. Thus, the intestinal microbiota not only contributes to the inflammatory environment that drives endometriosis but also plays a significant role in the formation of adhesions and the fertility issues associated with the condition.

Secondly, TNF-α plays a pivotal role in infertility. As an immunomodulatory cytokine with diverse biological effects, TNF-α is primarily secreted by macrophages and T cells. In women with endometriosis and infertility, an imbalance in the intestinal microbiota leads to a substantial increase in TNF-α levels in the peritoneal fluid, exacerbating the inflammatory environment and contributing to reproductive dysfunction (Wang XM and Song, 2018). Under normal conditions, TNF-α plays a crucial role in various biological processes, including the regulation of reproductive endocrinology, hormone synthesis, pregnancy maintenance, male spermatogenesis, and sperm function. However, elevated concentrations of TNF-α have toxic effects that impair fertility. Specifically, high levels of TNF-α can directly damage the normal morphology of sperm, reduce sperm motility, and interfere with key stages of fertilization and implantation, ultimately compromising reproductive success (Li et al., 2006; Wang XM and Song, 2018). During conception, sperm and egg meet at the ampulla of the oviduct. Elevated levels of TNF-α in the abdominal fluid of women with endometriosis can negatively affect the gametes and fertilized eggs within the fallopian tubes, thereby compromising fertility. High TNF-α levels can also: (1) promote the production of maternal prohormone E2, interfere with the coagulation system, and facilitate the formation of blood clots in the fetal disc; (2) damage decidual blood vessels, causing vessel retraction and impeding the normal blood supply to embryonic and fetal tissues, potentially leading to tissue necrosis, abortion, and infertility; (3) High TNF-α levels also impair decidual blood vessels, causing blood vessel retraction, which disrupts the normal blood flow to embryonic and fetal tissues. This reduction in blood supply can lead to tissue necrosis, increasing the risk of miscarriage and infertility.

Last but not least, dysbiosis of the gut microbiota can lead to elevated circulating estrogen levels. The gut microbiota includes specific bacteria that influence estrogen metabolism by secreting β-glucuronidase, which uncouples estrogen from its conjugated form into active, free estrogen. This free estrogen is then reabsorbed into the body through the enterohepatic circulation, thereby participating in the regulation of circulating estrogen levels (Dabek et al., 2008; Baker et al., 2017). The activity of β-glucuronidase is influenced by both the density of the bacterial population and dietary factors. When the gut microbiota is dysregulated or the diet is high in fat, the activity of β-glucuronidase in the intestine increases. This leads to a higher conversion of conjugated estrogen into free estrogen, thereby raising the levels of free estrogen in the body (Kwa et al., 2016). Additionally, gut microbiota can synthesize estrogen-like compounds from dietary sources, further enhancing estrogenic effects in the body. These compounds can interact with estrogen receptors and mimic the actions of endogenous estrogens, potentially influencing various physiological processes related to estrogen regulation. Flores et al. (2012) studied men and postmenopausal women, both of whom do not have ovarian-origin estrogen, and found that urinary estrogen levels and most estrogen metabolites were closely linked to the richness and α-diversity of fecal microbiota (R ≥ 0.5, P ≤ 0.003). This suggests a strong connection between gut microbiota and estrogen metabolism. When the intestinal microbiota is disrupted, it can directly impact the body’s estrogen levels. Additionally, many aspects of female reproductive health, such as follicular growth, endometrial hyperplasia, endometrial receptivity, corpus luteum function, and early placental perfusion, are tightly regulated by estrogen. The pathological changes associated with endometriosis, which lead to gut microbiota imbalance, may, therefore, affect the normal metabolism of estrogen, ultimately impairing fertility in women.

However, there is a lack of direct studies linking endometriosis-induced gut microbiota imbalance to infertility. Several reasons contribute to this gap in research. First, it remains unclear which specific changes in the gut microbiota and to what extent these changes occur due to endometriosis, making it difficult to design complementary diagnostic tests. Furthermore, fertility is a complex and multifactorial process, and it is challenging to pinpoint a single pathway as the definitive cause of infertility.

### 3.6 Therapeutic approaches targeting gut microbiota

Probiotics, beneficial bacteria that promote gut health, have been shown to positively influence gut microbiota and modulate immune responses. In the context of endometriosis, specific strains of Lactobacillus and Bifidobacterium have emerged as promising candidates for reducing inflammation (Itoh et al., 2010). By strengthening the intestinal barrier, probiotics can help prevent endotoxins from entering the bloodstream, which in turn reduces systemic inflammation associated with endometriosis. They also assist in the metabolism of estrogen through the gut-liver axis, helping to lower excess estrogen that drives disease progression. In addition, specific probiotic strains can boost the activity of regulatory T cells, which are essential for tempering the overactive immune response commonly observed in endometriosis. By enhancing regulatory T cell function, these probiotics may help to moderate inflammation and immune dysregulation, offering a potential pathway for managing endometriosis-related symptoms. While clinical trials on the use of probiotics for endometriosis are still limited, preliminary studies in animals and small-scale human trials indicate potential benefits in alleviating both pain and inflammation.

Prebiotics, non-digestible fibers that support the growth of beneficial gut bacteria, have shown promise in positively affecting both the gut microbiome and immune responses. Compounds such as inulin, fructooligosaccharides (FOS), and galactooligosaccharides (GOS) can benefit individuals with endometriosis by encouraging the proliferation of Lactobacillus and Bifidobacterium species. This growth helps rebalance the gut microbiota, potentially reducing systemic inflammation and lowering estrogen levels. Additionally, prebiotics stimulate the production of short-chain fatty acids (SCFAs), such as butyrate, which possess anti-inflammatory properties and may aid in modulating immune responses associated with endometriosis (Chadchan et al., 2021). Synbiotics, a combination of probiotics and prebiotics, work together to enhance the survival and efficacy of beneficial microorganisms. This synergistic effect may be especially useful in managing endometriosis. By promoting a healthy gut microbiota, synbiotics help maintain microbial balance, reducing harmful bacteria while increasing beneficial strains, which can subsequently lower inflammation. Additionally, by regulating gut bacteria involved in estrogen metabolism, synbiotics may decrease estrogen reabsorption, potentially helping to curb estrogen-driven endometrial growth.

Fecal Microbiota Transplantation (FMT) is a procedure that involves transferring fecal microbiota from a healthy donor to a recipient to restore the balance of the gut microbiome. Although FMT is commonly used to treat Clostridioides difficile infections, its application in endometriosis is still experimental. This procedure has the potential to rebalance the gut microbiome, which may help reduce inflammation, correct dysbiosis, and enhance immune regulation (Que et al., 2024). By altering gut microbial populations, FMT could contribute to decreased systemic inflammation, a critical factor in the onset and progression of endometriosis. Diet significantly influences the composition of the gut microbiota, and dietary modifications that support gut health may provide therapeutic benefits (Brouns et al., 2023; Cirillo et al., 2023). Diets rich in omega-3 fatty acids, fiber, and polyphenols, —such as the Mediterranean diet—can positively impact gut microbiota and help mitigate inflammation (Brouns et al., 2023). These diets also enhance the production of short-chain fatty acids (SCFAs), which are important for regulating immune function and hormone metabolism (Cirillo et al., 2023). Conversely, reducing the intake of refined sugars, saturated fats, and processed foods may help prevent gut dysbiosis and potentially decrease inflammation linked to endometriosis. While antibiotics are frequently prescribed to treat bacterial infections, they can disrupt gut microbiota and may exacerbate dysbiosis when used indiscriminately. Therefore, microbiota-targeted therapies, which focus on eliminating harmful bacteria or enhancing beneficial strains, could offer a more precise strategy for managing endometriosis. Therapeutic approaches centered on the gut microbiota present promising new options for treating endometriosis. Although research in this area is still in its early stages, interventions such as probiotics, prebiotics, synbiotics, FMT, and dietary modifications show potential for reducing inflammation, modulating immune responses, and regulating estrogen metabolism—all crucial factors in the development of endometriosis. Future clinical trials will be essential to thoroughly evaluate the efficacy and safety of these microbiota-focused therapies in managing this condition.

Future research should emphasize the importance of conducting longitudinal studies to assess how changes in gut microbiota impact the onset and progression of endometriosis over time, thereby clarifying the causal links between gut dysbiosis and related symptoms. There is a pressing need for well-designed clinical trials to determine the safety and effectiveness of microbiota-based interventions—such as probiotics, prebiotics, synbiotics, and fecal microbiota transplantation (FMT)—in women suffering from endometriosis, with a focus on alleviating symptoms and enhancing overall quality of life and reproductive health. Furthermore, investigations should delve into the specific biological pathways through which gut microbiota affect endometrial health, including the roles of neuroinflammation, immune modulation, and estrogen metabolism. Given the psychological aspects associated with endometriosis, future studies should also explore the gut-brain axis in detail to understand how gut health influences mental well-being in affected women. In addition, personalized dietary interventions tailored to individual microbiome profiles should be explored to optimize gut health and mitigate endometriosis symptoms. Research should also consider how ethnic, genetic, and environmental differences influence gut microbiota composition and the associated risk of developing endometriosis. Lifestyle factors, including stress management, physical activity, and sleep patterns, should be investigated for their effects on gut microbiota and their potential contributions to the pathophysiology of endometriosis. Employing a multi-omics approach—incorporating genomics, transcriptomics, proteomics, and metabolomics—could provide valuable insights into the complex relationship between gut microbiota and endometriosis, possibly leading to new biomarkers for diagnosis and treatment. Finally, educational initiatives aimed at raising awareness of the impact of gut health on endometriosis are essential, empowering patients to make informed decisions regarding their dietary and lifestyle choices. Promoting interdisciplinary collaboration among researchers, healthcare providers, nutritionists, and mental health specialists will be crucial in developing comprehensive strategies that address both the physical and psychological dimensions of endometriosis.

## 4 Conclusion

The increasing number of endometriosis diagnoses is contributing to a significant public health burden, leading to challenges such as abdominal masses, pelvic pain, infertility, and associated psychological distress. Endometriosis disrupts metabolism, resulting in systemic inflammation and alterations in brain function that heighten pain perception and contribute to mood and anxiety disorders. This underscores the urgent need for a paradigm shift in the management of endometriosis—moving beyond conventional biomedical strategies to adopt a holistic approach that combines traditional medical treatments with psychological and nutritional interventions.

By fostering interdisciplinary collaboration and prioritizing patient-centered care, we can significantly enhance the quality of life and overall well-being of individuals affected by endometriosis. Modulating the gut microbiome to restore metabolic balance also presents an exciting therapeutic opportunity. However, it is essential to recognize the limitations of existing research and advocate for ongoing exploration into the long-term effectiveness and safety of emerging treatments. Understanding the socio-cultural factors that influence the experiences of those living with endometriosis. While recent advances in endometriosis research are promising, we must remain mindful of these limitations and emphasize the need for continued investigation.

## References

[B1] Agostinis C. Zorzet S. Balduit A. Zito G. Mangogna A. Macor P.. (2021). The inflammatory feed-forward loop triggered by the complement component C3 as a potential target in endometriosis. Front. Immunol. 12. doi: 10.3389/fimmu.2021.693118, PMID: 34489939 PMC8418148

[B2] Alghetaa H. Mohammed A. Singh N. P. Bloomquist R. F. Chatzistamou I. Nagarkatti M.. (2023). Estrobolome dysregulation is associated with altered immunometabolism in a mouse model of endometriosis. Front. Endocrinol. 14. doi: 10.3389/fendo.2023.1261781, PMID: 38144564 PMC10748389

[B3] Allaire C. Bedaiwy M. A. Yong P. J. (2023). Diagnosis and management of endometriosis. Can. Med. Assoc. J. 195, E363–E371. doi: 10.1503/cmaj.220637, PMID: 36918177 PMC10120420

[B4] Antsiferova Y. S. Sotnikova N. Y. Posiseeva L. V. Shor A. L. (2005). Changes in the T-helper cytokine profile and in lymphocyte activation at the systemic and local levels in women with endometriosis. Fertil. Steril. 84, 1705–1711. doi: 10.1016/j.fertnstert.2005.05.066, PMID: 16359969

[B5] As-Sanie S. Harris R. E. Napadow V. Kim J. Neshewat G. Kairys A.. (2012). Changes in regional gray matter volume in women with chronic pelvic pain: A voxel-based morphometry study. Pain 153, 1006–1014. doi: 10.1016/j.pain.2012.01.032, PMID: 22387096 PMC3613137

[B6] As-Sanie S. Kim J. Schmidt-Wilcke T. Clauw D. J. Napadow V. Harris R. E.. (2016). Functional connectivity is associated with altered brain chemistry in women with endometriosis-associated chronic pelvic pain. J. Pain. 17, 1–13. doi: 10.1016/j.jpain.2015.09.008, PMID: 26456676 PMC4698023

[B7] Ata B. Yildiz S. Turkgeldi E. Brocal V. P. Dinleyici E. C. Moya A.. (2019). The endobiota study: comparison of vaginal, cervical and gut microbiota between women with stage 3/4 endometriosis and healthy controls. Sci. Rep. 9, 2204. doi: 10.1038/s41598-019-39700-6, PMID: 30778155 PMC6379373

[B8] Bailey M. T. Coe C. (2002). Endometriosis is associated with an altered profile of intestinal microflora in female rhesus monkeys. Hum. Reprod. 17, 5. doi: 10.1093/humrep/17.7.1704, PMID: 12093827

[B9] Baker J. M. Al-Nakkash L. Herbst-Kralovetz M. M. (2017). Estrogen–gut microbiome axis: Physiological and clinical implications. Maturitas 103, 45–53. doi: 10.1016/j.maturitas.2017.06.025, PMID: 28778332

[B10] Brouns F. Van Haaps A. Keszthelyi D. Venema K. Bongers M. Maas J.. (2023). Diet associations in endometriosis: a critical narrative assessment with special reference to gluten. Front. Nutr. 10. doi: 10.3389/fnut.2023.1295983, PMID: 37731404 PMC10507348

[B11] Bulun S. E. Yilmaz B. D. Sison C. Miyazaki K. Bernardi L. Liu S.. (2019). Endometriosis. Endocr. Rev. 40, 1048–1079. doi: 10.1210/er.2018-00242, PMID: 30994890 PMC6693056

[B12] Cao Y. Jiang C. Jia Y. Xu D. Yu Y. (2020). Letrozole and the traditional chinese medicine, shaofu zhuyu decoction, reduce endometriotic disease progression in rats: A potential role for gut microbiota. Evid. Based. Complement. Alternat. Med. 2020, 3687498. doi: 10.1155/2020/3687498, PMID: 32765629 PMC7387974

[B13] Chadchan S. B. Cheng M. Parnell L. A. Yin Y. Schriefer A. Mysorekar I. U.. (2019). Antibiotic therapy with metronidazole reduces endometriosis disease progression in mice: a potential role for gut microbiota. Hum. Reprod. 34, 1106–1116. doi: 10.1093/humrep/dez041, PMID: 31037294 PMC6554192

[B14] Chadchan S. B. Naik S. K. Popli P. Talwar C. Putluri S. Ambati C. R.. (2023). Gut microbiota and microbiota-derived metabolites promotes endometriosis. Cell Death Discov. 9(1), 28. doi: 10.1038/s41420-023-01309-0, PMID: 36693853 PMC9873805

[B15] Chadchan S. B. Popli P. Ambati C. R. Tycksen E. Han S. J. Bulun S. E.. (2021). Gut microbiota–derived short-chain fatty acids protect against the progression of endometriosis. Life Sci. Alliance. 4(12), e202101224. doi: 10.26508/lsa.202101224, PMID: 34593556 PMC8500332

[B16] Chang K. K. Liu L. B. Jin L. P. Zhang B. Mei J. Li H.. (2017). IL-27 triggers IL-10 production in Th17 cells via a c-Maf/RORγt/Blimp-1 signal to promote the progression of endometriosis. Cell Death Dis. 8, e2666–e2666. doi: 10.1038/cddis.2017.95, PMID: 28300844 PMC5386585

[B17] Chen C. Song X. Wei W. Zhong H. Dai J. Lan Z.. (2017). The microbiota continuum along the female reproductive tract and its relation to uterine-related diseases. Nat. Commun. 8(1), 875. doi: 10.1038/s41467-017-00901-0, PMID: 29042534 PMC5645390

[B18] Chen S. Liu Y. Zhong Z. Wei C. Liu Y. Zhu X.. (2023). Peritoneal immune microenvironment of endometriosis: Role and therapeutic perspectives. Front. Immunol. 14. doi: 10.3389/fimmu.2023.1134663, PMID: 36865552 PMC9971222

[B19] Chen Y. Wang K. Xu Y. Guo P. Hong B. Cao Y. (2019). Alteration of myeloid-derived suppressor cells, chronic inflammatory cytokines, and exosomal miRNA contribute to the peritoneal immune disorder of patients with endometriosis. Reprod. Sci. 26, 1130–1138. doi: 10.1177/1933719118808923, PMID: 30453861

[B20] Cirillo M. Argento F. R. Becatti M. Fiorillo C. Coccia M. E. Fatini C. (2023). Mediterranean diet and oxidative stress: A relationship with pain perception in endometriosis. Int. J. Mol. Sci. 24(19), 14601. doi: 10.3390/ijms241914601, PMID: 37834048 PMC10572576

[B21] Cordeiro M. R. Carvalhos C. A. Figueiredo-Dias M. (2022). The emerging role of menstrual-blood-derived stem cells in endometriosis. Biomedicines 11(1), 39. doi: 10.3390/biomedicines11010039, PMID: 36672546 PMC9856091

[B22] Cuffaro F. Russo E. Amedei A. (2024). Endometriosis, pain, and related psychological disorders: unveiling the interplay among the microbiome, inflammation, and oxidative stress as a common thread. Int. J. Mol. Sci. 25(12), 6473. doi: 10.3390/ijms25126473, PMID: 38928175 PMC11203696

[B23] Dabek M. McCrae S. I. Stevens V. J. Duncan S. H. Louis P. (2008). Distribution of β-glucosidase and β-glucuronidase activity and of β-glucuronidase gene gus in human colonic bacteria. FEMS Microbiol. Ecol. 66, 487–495. doi: 10.1111/j.1574-6941.2008.00520.x, PMID: 18537837

[B24] de Barros I. B. L. Malvezzi H. Gueuvoghlanian-Silva B. Y. Piccinato C. A. Rizzo L. V. Podgaec S.. (2017). What do we know about regulatory T cells and endometriosis? A systematic review. J. Reprod. Immunol. 120, 48–55. doi: 10.1016/j.jri.2017.04.003, PMID: 28463710

[B25] Ding A. Noga H. Bouchard K. N. Bedaiwy M. A. Lee C. Allaire C.. (2024). Pain with orgasm in endometriosis: potential etiologic factors and clinical correlates. J. Sexual. Med. 21, 807–815. doi: 10.1093/jsxmed/qdae084, PMID: 39039031 PMC11372072

[B26] Do H. Diaz-Sylvester P. Groesch K. Wilson T. Delfino K. de Mola J. R. L.. (2024). Influence of hormonal factors, number of sexual partners, surgical intervention on gastrointestinal and urogenital microbiota of patients endometriosis. Arch. Med. Res. 55, 103112. doi: 10.1016/j.arcmed.2024.103112, PMID: 39500248

[B27] Drury J. A. Parkin K. L. Coyne L. Giuliani E. Fazleabas A. T. Hapangama D.K. (2018). The dynamic changes in the number of uterine natural killer cells are specific to the eutopic but not to the ectopic endometrium in women and in a baboon model of endometriosis. Reprod. Biol. Endocrinol. 16(1), 67. doi: 10.1186/s12958-018-0385-3, PMID: 30021652 PMC6052567

[B28] Duan J. Liu X. Wang H. Guo S. W. (2018). The M2a macrophage subset may be critically involved in the fibrogenesis of endometriosis in mice. Reprod. BioMedi. Online. 37, 254–268. doi: 10.1016/j.rbmo.2018.05.017, PMID: 30314882

[B29] Eşkin Tanrıverdi M. D. Kaya Sezginer E. Erol Koç E. M. Moraloğlu Tekin Ö. (2025). Evaluation of serum and peritoneal fluid mannose-binding lectin associated serine protease-3, adipsin, properdin, and complement factor-H levels in endometriosis patients. Int. J. Gynecol. Obstet. doi: 10.1002/ijgo.16195, PMID: 39907303

[B30] Fan D. Wang X. Shi Z. Jiang Y. Zheng B. Xu L.. (2023). Understanding endometriosis from an immunomicroenvironmental perspective. Chin. Med. J. 136(16), 1897–1909. doi: 10.1097/CM9.0000000000002649, PMID: 37439327 PMC10431529

[B31] Fasano A. Chassaing B. Haller D. Flores Ventura E. Carmen-Collado M. Pastor N.. (2024). Microbiota during pregnancy and early life: role in maternal–neonatal outcomes based on human evidence. Gut. Microbes 16(1), 2392009. doi: 10.1080/19490976.2024.2392009, PMID: 39161102 PMC11340748

[B32] Flores R. S. J. Fuhrman B. Xu X. Veenstra T. D. Gail M. H. Gajer P.. (2012). Fecal microbial determinants of fecal and systemic estrogens and estrogen metabolites: a cross-sectional study. J. Transl. Med. 10, 11. doi: 10.1186/1479-5876-10-253, PMID: 23259758 PMC3552825

[B33] Fonseca M. A. S. Haro M. Wright K. N. Lin X. Abbasi F. Sun J.. (2023). Single-cell transcriptomic analysis of endometriosis. Nat. Genet. 55, 255–267. doi: 10.1038/s41588-022-01254-1, PMID: 36624343 PMC10950360

[B34] Frodl T. O’Keane V. (2013). How does the brain deal with cumulative stress? A review with focus on developmental stress, HPA axis function and hippocampal structure in humans. Neurobiol. Dis. 52, 24–37. doi: 10.1016/j.nbd.2012.03.012, PMID: 22426398

[B35] Gete D. G. Doust J. Mortlock S. Montgomery G. Mishra G. D. (2023). Associations between endometriosis and common symptoms: findings from the Australian Longitudinal Study on Women’s Health. Am. J. Obstet. Gynecol. 229, 536.e531–536.e520. doi: 10.1016/j.ajog.2023.07.033, PMID: 37499990

[B36] Gogacz M. Winkler I. Bojarska-Junak A. Tabarkiewicz J. Semczuk A. Rechberger T.. (2016). Increased percentage of Th17 cells in peritoneal fluid is associated with severity of endometriosis. J. Reprod. Immunol. 117, 39–44. doi: 10.1016/j.jri.2016.04.289, PMID: 27371900

[B37] Hantschel J. Weis S. Schäfer K. H. Menger M. D. Kohl M. Egert M.. (2019). Effect of endometriosis on the fecal bacteriota composition of mice during the acute phase of lesion formation. PloS One 14, e0226835. doi: 10.1371/journal.pone.0226835, PMID: 31887116 PMC6936831

[B38] Hicks C. Leonardi M. Chua X. Y. Mari-Breedt L. Espada M. El-Omar E. M.. (2024). Oral, vaginal, and stool microbial signatures in patients with endometriosis as potential diagnostic non-invasive biomarkers: A prospective cohort study. BJOG. 132(3), 326–336. doi: 10.1111/1471-0528.17979, PMID: 39431364 PMC11704027

[B39] Horne A. W. Missmer S. A. (2022). Pathophysiology, diagnosis, and management of endometriosis. Bmj. 379, e070750. doi: 10.1136/bmj-2022-070750, PMID: 36375827

[B40] Huang L. Liu B. Liu Z. Feng W. Liu M. Wang Y.. (2021). Gut microbiota exceeds cervical microbiota for early diagnosis of endometriosis. Front. Cell. Infect. Microbiol. 11. doi: 10.3389/fcimb.2021.788836, PMID: 34950610 PMC8688745

[B41] Itoh H. Uchida M. Sashihara T. Ji Z. S. Li J. Tang Q.. (2010). Lactobacillus gasseri OLL2809 is effective especially on the menstrual pain and dysmenorrhea in endometriosis patients: randomized, double-blind, placebo-controlled study. Cytotechnology 63, 153–161. doi: 10.1007/s10616-010-9326-5, PMID: 21153437 PMC3080472

[B42] Jensen K. B. Regenbogen C. Ohse M. C. Frasnelli J. Freiherr J. Lundström J. N. (2016). Brain activations during pain. Pain 157, 1279–1286. doi: 10.1097/j.pain.0000000000000517, PMID: 26871535

[B43] Ji R. R. Nackley A. Huh Y. Terrando N. Maixner W. (2018). Neuroinflammation and central sensitization in chronic and widespread pain. Anesthesiology 129, 343–366. doi: 10.1097/ALN.0000000000002130, PMID: 29462012 PMC6051899

[B44] Jiang I. Yong P.J. Allaire C. Bedaiwy M. A. (2021). Intricate connections between the microbiota and endometriosis. Int. J. Mol. Sci. 22(11), 5644. doi: 10.3390/ijms22115644, PMID: 34073257 PMC8198999

[B45] Jimenez N. Norton T. Diadala G. Bell E. Valenti M. Farland L. V.. (2024). Vaginal and rectal microbiome contribute to genital inflammation in chronic pelvic pain. BMC Med. 22(1), 283. doi: 10.1186/s12916-024-03500-1, PMID: 38972981 PMC11229265

[B46] Kabut J. Kondera-Anasz Z. Sikora J. Mielczarek-Palacz A. (2007). Levels of complement components iC3b, C3c, C4, and SC5b-9 in peritoneal fluid and serum of infertile women with endometriosis. Fertil. Steril. 88, 1298–1303. doi: 10.1016/j.fertnstert.2006.12.061, PMID: 17482181

[B47] Karadadas E. Hortu I. Ak H. Ergenoglu A. M. Karadadas N. Aydin H. H. (2020). Evaluation of complement system proteins C3a, C5a and C6 in patients of endometriosis. Clin. Biochem. 81, 15–19. doi: 10.1016/j.clinbiochem.2020.04.005, PMID: 32325082

[B48] Kim C.-S. (2024). Roles of diet-associated gut microbial metabolites on brain health: cell-to-cell interactions between gut bacteria and the central nervous system. Adv. Nutr. 15(1), 100136. doi: 10.1016/j.advnut.2023.10.008, PMID: 38436218 PMC10694655

[B49] Koninckx P. R. Ussia A. Adamyan L. Wattiez A. Gomel V. Martin D. C. (2019). Pathogenesis of endometriosis: the genetic/epigenetic theory. Fertil. Steril. 111, 327–340. doi: 10.1016/j.fertnstert.2018.10.013, PMID: 30527836

[B50] Kulkoyluoglu-Cotul E. Arca A. Madak-Erdogan Z. (2019). Crosstalk between estrogen signaling and breast cancer metabolism. Trends Endocrinol. Metab. 30, 25–38. doi: 10.1016/j.tem.2018.10.006, PMID: 30471920

[B51] Kwa M. P. C. Blaser M. J. Adams S. (2016). The intestinal microbiome and estrogen receptor–positive female breast cancer. J. Natl. Cancer Inst. 108, 10. doi: 10.1093/jnci/djw029, PMID: 27107051 PMC5017946

[B52] Laganà A. S. La Rosa V.L. Rapisarda A. M. C. Valenti G. Sapia F. Chiofalo B.. (2017). Anxiety and depression in patients with endometriosis: impact and management challenges. Int. J. Women’s. Health 9, 323–330. doi: 10.2147/IJWH.S119729, PMID: 28553145 PMC5440042

[B53] Lamceva J. Uljanovs R. Strumfa I. (2023). The main theories on the pathogenesis of endometriosis. Int. J. Mol. Sci. 24(5), 4254. doi: 10.3390/ijms24054254, PMID: 36901685 PMC10001466

[B54] Le N. Cregger M. Brown V. Loret de Mola J. Bremer P. Nguyen L.. (2021). Association of microbial dynamics with urinary estrogens and estrogen metabolites in patients with endometriosis. PloS One 16, e0261362. doi: 10.1371/journal.pone.0261362, PMID: 34914785 PMC8675749

[B55] Le N. Cregger M. Fazleabas A. Braundmeier-Fleming A. (2022). Effects of endometriosis on immunity and mucosal microbial community dynamics in female olive baboons. Sci. Rep. 12, 1590. doi: 10.1038/s41598-022-05499-y, PMID: 35102185 PMC8803974

[B56] Lee G. J. Porreca F. Navratilova E. (2023). Prolactin and pain of endometriosis. Pharmacol. Ther. 247, 10843. doi: 10.1016/j.pharmthera.2023.108435, PMID: 37169264 PMC12181984

[B57] Li M. W. Xia W. Mruk D. D. Wang C. Q. Yan H. H. Siu M. K.. (2006). Tumor necrosis factor α reversibly disrupts the blood–testis barrier and impairs Sertoli–germ cell adhesion in the seminiferous epithelium of adult rat testes. J. Endocrinol. 190, 313–329. doi: 10.1677/joe.1.06781, PMID: 16899565

[B58] Li Q. Yuan M. Jiao X. Ji M. Huang Y. Li J.. (2021). Metabolite profiles in the peritoneal cavity of endometriosis patients and mouse models. Reprod. BioMedi. Online. 43, 810–819. doi: 10.1016/j.rbmo.2021.06.029, PMID: 34538753

[B59] Li Y. Zhou Z. Liang X. Ding J. He Y. Sun S.. (2024). Gut microbiota disorder contributes to the production of IL-17A that exerts chemotaxis via binding to IL-17RA in endometriosis. J. Inflammation Res. 17, 4199–4217. doi: 10.2147/JIR.S458928, PMID: 38974001 PMC11225878

[B60] Liu H. Li J. Guan C. Gao W. Li Y. Wang J.. (2024). Endometriosis is a disease of immune dysfunction, which could be linked to microbiota. Front. Genet. 15. doi: 10.3389/fgene.2024.1386411, PMID: 38974388 PMC11227297

[B61] Liu Y. Y. Liu Y. Hu W. T. Tang L. L. Sheng Y. R. Wei C. Y.. (2019). Elevated heme impairs macrophage phagocytosis in endometriosis. Reproduction 158, 10. doi: 10.1530/REP-19-0028, PMID: 31299634

[B62] Liu J. Yang D. Piao C. Wang X. Sun X. Li Y.. (2023). UPLC-Q-TOF/MS based plasma metabolomics for identification of paeonol’s metabolic target in endometriosis. Molecules 28(2), 653. doi: 10.3390/molecules28020653, PMID: 36677710 PMC9864815

[B63] Maddern J. Grundy L. Castro J. Brierley S. M. (2020). Pain in endometriosis. Front. Cell. Neurosci. 14. doi: 10.3389/fncel.2020.590823, PMID: 33132854 PMC7573391

[B64] Martire F. G. Piccione E. Exacoustos C. Zupi E. (2023). Endometriosis and adolescence: the impact of dysmenorrhea. J. Clin. Med. 12(17), 5624. doi: 10.3390/jcm12175624, PMID: 37685691 PMC10488856

[B65] McKinnon B. Bersinger N. A. Wotzkow C. Mueller M. D. (2012). Endometriosis-associated nerve fibers, peritoneal fluid cytokine concentrations, and pain in endometriotic lesions from different locations. Fertil. Steril. 97, 373–380. doi: 10.1016/j.fertnstert.2011.11.011, PMID: 22154765

[B66] Meek S. C. Hodge D. Musich J. R. (1988). Autoimmunity in infertile patients with endometriosis. Am. J. Obstet. Gynecol. 158, 9. doi: 10.1016/0002-9378(88)90369-9, PMID: 3132855

[B67] Munrós J. Tàssies D. Reverter J.C. Martin L. Pérez A. Carmona F.. (2019). Circulating neutrophil extracellular traps are elevated in patients with deep infiltrating endometriosis. Reprod. Sci. 26, 70–76. doi: 10.1177/1933719118757682, PMID: 29448896

[B68] Muraoka A. S. M. Hamaguchi T. Watanabe S. Iijima K. Murofushi Y. Shinjo K.. (2023). Fusobacterium infection facilitates the development of endometriosis through the phenotypic transition of endometrial fibroblasts. Sci. Transl. Med. 15, 18. doi: 10.1126/scitranslmed.add1531, PMID: 37315109

[B69] Nassiri Kigloo H. Itani R. Montreuil T. Feferkorn I. Raina J. Tulandi T.. (2024). Endometriosis, chronic pain, anxiety, and depression: A retrospective study among 12 million women. J. Affect. Disord. 346, 260–265. doi: 10.1016/j.jad.2023.11.034, PMID: 37956828

[B70] Nati I. D. Malutan A. Ciortea R. Oancea M. Bucuri C. Roman M.. (2024). Exploring the influence of IL-8, IL-10, patient-reported pain, and physical activity on endometriosis severity. Diagnostics 14(16), 1822. doi: 10.3390/diagnostics14161822, PMID: 39202309 PMC11353965

[B71] Nezhat C. Khoyloo F. Tsuei A. Armani E. Page B. Rduch T.. (2024). The prevalence of endometriosis in patients with unexplained infertility. J. Clin. Med. 13(2), 444. doi: 10.3390/jcm13020444, PMID: 38256580 PMC11326441

[B72] Ni Y. Hu L. Yang S. Ni L. Ma L. Zhao Y.. (2021). Bisphenol A impairs cognitive function and 5-HT metabolism in adult male mice by modulating the microbiota-gut-brain axis. Chemosphere 282, 130952. doi: 10.1016/j.chemosphere.2021.130952, PMID: 34082316

[B73] Ni Z. Sun S. Bi Y. Ding J. Cheng W. Yu J.. (2020). Correlation of fecal metabolomics and gut microbiota in mice with endometriosis. Am. J. Reprod. Immunol. 84, e13307. doi: 10.1111/aji.13307, PMID: 32681566

[B74] Nijs J. M. A. Ickmans K. Baert I. Meeus M. (2014). Treatment of central sensitization in patients with ‘unexplained’ chronic pain: an update. Expert Opin. Pharmacother. 15, 13. doi: 10.1517/14656566.2014.925446, PMID: 24930805

[B75] Oală I. E. Mitranovici M. I. Chiorean D. M. Irimia T. Crișan A. I. Melinte I. M.. (2024). Endometriosis and the role of pro-inflammatory and anti-inflammatory cytokines in pathophysiology: A narrative review of the literature. Diagnostics 14(3), 312. doi: 10.3390/diagnostics14030312, PMID: 38337827 PMC10855755

[B76] Ochoa Bernal M. A. Fazleabas A. T. (2024). The known, the unknown and the future of the pathophysiology of endometriosis. Int. J. Mol. Sci. 25(11), 5815. doi: 10.3390/ijms25115815, PMID: 38892003 PMC11172035

[B77] Osuga Y. Koga K. Hirota Y. Hirata T. Yoshino O. Taketani Y. (2011). Lymphocytes in endometriosis. Am. J. Reprod. Immunol. 65, 1–10. doi: 10.1111/j.1600-0897.2010.00887.x, PMID: 20584009

[B78] Ota H. Maki M. Shidara Y. Kodama H. Takahashi H. Hayakawa M.. (1992). Effects of danazol at the immunologic level in patients with adenomyosis, with special reference to autoantibodies: A multi-center cooperative study. Am. J. Obstet. Gynecol. 167, 481–486. doi: 10.1016/S0002-9378(11)91433-1, PMID: 1497054

[B79] Pai A. H. Wang Y. W. Lu P. C. Wu H. M. Xu J. L. Huang H. Y. (2023). Gut microbiome–estrobolome profile in reproductive-age women with endometriosis. Int. J. Mol. Sci. 24(22), 16301. doi: 10.3390/ijms242216301, PMID: 38003489 PMC10671785

[B80] Pant A. Moar K. Arora K. T. Maurya P. K. (2023). Biomarkers of endometriosis. Clin. Chim. Acta. 549, 117563. doi: 10.1016/j.cca.2023.117563, PMID: 37739024

[B81] Patel B. G. Rudnicki M. Yu J. Shu Y. Taylor R. N. (2017). Progesterone resistance in endometriosis: origins, consequences and interventions. Acta Obstet. Gynecol. Scand. 96, 623–632. doi: 10.1111/aogs.13156, PMID: 28423456

[B82] Pérez-Prieto I. Vargas E. Salas-Espejo E. Lüll K. Canha-Gouveia A. Pérez L. A.. (2024). Gut microbiome in endometriosis: a cohort study on 1000 individuals. BMC Med. 22(1), 294. doi: 10.1186/s12916-024-03503-y, PMID: 39020289 PMC11256574

[B83] Podgaec S. Abrao M. S. Dias J. A., Jr. Rizzo L. V. de Oliveira R. M. Baracat E. C. (2007) Endometriosis: an inflammatory disease with a Th2 immune response comp onent. Hum. Reprod. (Oxford. England). 22, 1373–1379. doi: 10.1093/humrep/del516, PMID: 17234676

[B84] Que M. Li S. Xia Q. Li X. Luo X. Zhan G.. (2024). Microbiota-gut-brain axis in perioperative neurocognitive and depressive disorders: Pathogenesis to treatment. Neurobiol. Dis. 200, 106627. doi: 10.1016/j.nbd.2024.106627, PMID: 39111702

[B85] Quinn M. J. (2011). Endometriosis: the consequence of uterine denervation–reinnervation. Arch. Gynecol. Obstet. 284, 1423–1429. doi: 10.1007/s00404-011-2063-y, PMID: 21932088

[B86] Raffaelli B. Overeem L.H. Mecklenburg J. Hofacker M. D. Knoth H. Nowak C. P.. (2021). Plasma calcitonin gene-related peptide (CGRP) in migraine and endometriosis during the menstrual cycle. Ann. Clin. Trans. Neurol. 8, 1251–1259. doi: 10.1002/acn3.51360, PMID: 33934575 PMC8164854

[B87] Rahal D. Bezerra Sobrinho C. Vilas Boas L. Capellari C. A. Andrade F. A. Nisihara R. (2023). C5a serum levels in patients with endometriosis: A cross-sectional study. Immunol. Invest. 52, 561–566. doi: 10.1080/08820139.2023.2206436, PMID: 37129467

[B88] Ramírez-Pavez T. N. Martínez-Esparza M. Ruiz-Alcaraz A. J. Marín-Sánchez P. Machado-Linde F. García-Peñarrubia P.. (2021). The role of peritoneal macrophages in endometriosis. Int. J. Mol. Sci. 22(19), 10792. doi: 10.3390/ijms221910792, PMID: 34639133 PMC8509388

[B89] Reis J. L. Rosa N.N. Martins C. Ângelo-Dias M. Borrego L. M. Lima J. (2024). The role of NK and T cells in endometriosis. Int. J. Mol. Sci. 25, 10141. doi: 10.3390/ijms251810141, PMID: 39337624 PMC11432446

[B90] Riccio L. G. C. Jeljeli M. Santulli P. Chouzenoux S. Doridot L. Nicco C.. (2019). B lymphocytes inactivation by Ibrutinib limits endometriosis progression in mice. Hum. Reprod. 34, 1225–1234. doi: 10.1093/humrep/dez071, PMID: 31247078

[B91] Riccio L. Santulli P. Marcellin L. Abrão M. S. Batteux F. Chapron C. (2018). Immunology of endometriosis. Best Pract. Res. Clin. Obstet. Gynaecol. 50, 39–49. doi: 10.1093/humrep/dez071, PMID: 29506962

[B92] Round J. L. Mazmanian S. K. (2009). The gut microbiota shapes intestinal immune responses during health and disease. Nat. Rev. Immunol. 9, 313–323. doi: 10.1038/nri2515, PMID: 19343057 PMC4095778

[B93] Salliss M. E. Farland L. V. Mahnert N. D. Herbst-Kralovetz M. M. (2022). The role of gut and genital microbiota and the estrobolome in endometriosis, infertility and chronic pelvic pain. Hum. Reprod. Update. 28, 92–131. doi: 10.1093/humupd/dmab035, PMID: 34718567

[B94] Samimi M. Pourhanifeh M. H. Mehdizadehkashi A. Eftekhar T. Asemi Z. (2019). The role of inflammation, oxidative stress, angiogenesis, and apoptosis in the pathophysiology of endometriosis: Basic science and new insights based on gene expression. J. Cell. Physiol. 234, 19384–19392. doi: 10.1002/jcp.28666, PMID: 31004368

[B95] Sampson J. A. (1927). Metastatic or Embolic Endometriosis, due to the Menstrual Dissemination of Endometrial Tissue into the Venous Circulation. Am. J. Pathol. 3(2), 93–110. 19969738 PMC1931779

[B96] Sarsenova M. Lawarde A. Pathare A. D. S. Saare M. Modhukur V. Soplepmann P.. (2024). Endometriotic lesions exhibit distinct metabolic signature compared to paired eutopic endometrium at the single-cell level. Commun. Biol. 7(1), 1026. doi: 10.1038/s42003-024-06713-5, PMID: 39169201 PMC11339455

[B97] Sasamoto N. Zeleznik O. A. Vitonis A. F. Missmer S. A. Laufer M. R. Avila-Pacheco J.. (2022). Presurgical blood metabolites and risk of postsurgical pelvic pain in young patients with endometriosis. Fertil. Steril. 117, 1235–1245. doi: 10.1016/j.fertnstert.2022.02.012, PMID: 35367064 PMC9149031

[B98] Saunders P. T. K. Horne A. W. (2021). Endometriosis: Etiology, pathobiology, and therapeutic prospects. Cell 184, 2807–2824. doi: 10.1016/j.cell.2021.04.041, PMID: 34048704

[B99] Saunders P. T. K. Whitaker L. H. R. Horne A. W. (2024). Endometriosis: Improvements and challenges in diagnosis and symptom management. Cell Rep. Med. 5(6), 101596. doi: 10.1016/j.xcrm.2024.101596, PMID: 38897171 PMC11228648

[B100] Shan J. Ni Z. Cheng W. Zhou L. Zhai D. Sun S.. (2021). Gut microbiota imbalance and its correlations with hormone and inflammatory factors in patients with stage 3/4 endometriosis. Arch. Gynecol. Obstet. 304, 1363–1373. doi: 10.1007/s00404-021-06057-z, PMID: 33839907

[B101] Shi J. L. Zheng Z. M. Chen M. Shen H. H. Li M. Q. Shao J. (2022). IL-17: an important pathogenic factor in endometriosis. Int. J. Med. Sci. 19, 769–778. doi: 10.7150/ijms.71972, PMID: 35582411 PMC9108413

[B102] Sikora J W-CA Smycz-Kubańska M. Mielczarek-Palacz A. Cygal A. Witek A. Kondera-Anasz Z. (2018). The role of complement components C1q, MBL and C1 inhibitor in pathogenesis of endometriosis. Arch. Gynecol. Obstet. 297, 7. doi: 10.1007/s00404-018-4754-0, PMID: 29572748 PMC5945730

[B103] Sikora J. Smycz-Kubańska M. Mielczarek-Palacz A. Kondera-Anasz Z. (2017) Abnormal peritoneal regulation of chemokine activation—The role of IL-8 in pathogenesis of endometriosis. Am. J. Reprod. Immunol. 77. doi: 10.1111/aji.12622, PMID: 28120482

[B104] Sinai D. Avni C. Toren P. (2024). Beyond physical pain: A large-scale cohort study on endometriosis trends and mental health correlates. J. Psychosom. Res. 182, 111809. doi: 10.1016/j.jpsychores.2024.111809, PMID: 38795400

[B105] Sisnett D. J. Zutautas K. B. Miller J. E. Lingegowda H. Ahn S. H. McCallion A.. (2024) The dysregulated IL-23/TH17 axis in endometriosis pathophysiology. J. Immunol. 212, 1428–1441. doi: 10.4049/jimmunol.2400018, PMID: 38466035

[B106] Smolarz B. Szyłło K. Romanowicz H. (2021). Endometriosis: epidemiology, classification, pathogenesis, treatment and genetics (Review of literature). Int. J. Mol. Sci. 22(19), 10554. doi: 10.3390/ijms221910554, PMID: 34638893 PMC8508982

[B107] Spinoni M. Porpora M. G. Muzii L. Grano C. (2024). Pain severity and depressive symptoms in endometriosis patients: mediation of negative body awareness and interoceptive self-regulation. J. Pain. 25(11), 104640. doi: 10.1016/j.jpain.2024.104640, PMID: 39032583

[B108] Stanic A. K. Kim M. Styer A. K. Rueda B. R. (2014). Dendritic cells attenuate the early establishment of endometriosis-like lesions in a murine model. Reprod. Sci. 21, 9. doi: 10.1177/1933719114525267, PMID: 24594835

[B109] Su C. Wan S. Ding J. Ni G. Ding H. (2024). Blood lipids mediate the effects of gut microbiome on endometriosis: a mendelian randomization study. Lipids Health Dis. 23(1), 110. doi: 10.1186/s12944-024-02096-y, PMID: 38627726 PMC11020997

[B110] Suen J. L. Chang Y. Shiu Y. S. Hsu C. Y. Sharma P. Chiu C. C.. (2019). IL-10 from plasmacytoid dendritic cells promotes angiogenesis in the early stage of endometriosis. J. Pathol. 249, 485–497. doi: 10.1002/path.5339, PMID: 31418859 PMC6899974

[B111] Suryawanshi S. Huang X. Elishaev E. Budiu R. A. Zhang L. Kim S.. (2014). Complement pathway is frequently altered in endometriosis and endometriosis-associated ovarian cancer. Clin. Cancer Res. 20, 6163–6174. doi: 10.1158/1078-0432.CCR-14-1338, PMID: 25294912 PMC4252715

[B112] Svensson A. Brunkwall L. Roth B. Orho-Melander M. Ohlsson B. (2021). Associations between endometriosis and gut microbiota. Reprod. Sci. 28, 2367–2377. doi: 10.1007/s43032-021-00506-5, PMID: 33660232 PMC8289757

[B113] Takamura M. Koga K. Izumi G. Urata Y. Nagai M. Hasegawa A.. (2016). Neutrophil depletion reduces endometriotic lesion formation in mice. Am. J. Reprod. Immunol. 76, 193–198. doi: 10.1111/aji.12540, PMID: 27432477

[B114] Tal A. Tal R. Pluchino N. Taylor H. S. (2019). Endometrial cells contribute to preexisting endometriosis lesions in a mouse model of retrograde menstruation†. Biol. Reprod. 100, 1453–1460. doi: 10.1093/biolre/ioz039, PMID: 30869747 PMC6561859

[B115] Talwar C. Singh V. Kommagani R. (2022). The gut microbiota: a double-edged sword in endometriosis. Biol. Reprod. 107(4), 881–901. doi: 10.1093/biolre/ioac147, PMID: 35878972 PMC9562115

[B116] Taylor H. S. Kotlyar A. M. Flores V. A. (2021). Endometriosis is a chronic systemic disease: clinical challenges and novel innovations. Lancet 397, 839–852. doi: 10.1016/S0140-6736(21)00389-5, PMID: 33640070

[B117] Thiel P. S. Bougie O. Pudwell J. Shellenberger J. Velez M. P. Murji A. (2024). Endometriosis and mental health: a population-based cohort study. Am. J. Obstet. Gynecol. 230, 649.e641–649.e619. doi: 10.1016/j.ajog.2024.01.023, PMID: 38307469

[B118] Toffoli M. Campisciano G. Santin A. Pegoraro S. Zito G. Spedicati B.. (2025). A possible association between low MBL/lectin pathway functionality and microbiota dysbiosis in endometriosis patients. Life Sci. 364, 123427. doi: 10.1016/j.lfs.2025.123427, PMID: 39892863

[B119] Troncon J. K. Zani A. C. Vieira A. D. Poli-Neto O. B. Nogueira A.A. Rosa-E-Silva J.C. (2014). Endometriosis in a patient with mayer-rokitansky-küster-hauser syndrome. Case Rep. Obstet. Gynecol. 2014, 1–4. doi: 10.1155/2014/376231, PMID: 25610677 PMC4293785

[B120] Tu C. H. Niddam D. M. Yeh T. C. Lirng J. F. Cheng C. M. Chou C. C.. (2013). Menstrual pain is associated with rapid structural alterations in the brain. Pain 154, 1718–1724. doi: 10.1016/j.pain.2013.05.022, PMID: 23693160

[B121] Tulandi T. Vercellini P. (2024). Growing evidence that endometriosis is a systemic disease. Reprod. BioMedi. Online. 49(3), 104292. doi: 10.1016/j.rbmo.2024.104292, PMID: 38943810

[B122] Ustianowska K. Ustianowski Ł. Machaj F. Gorący A. Rosik J. Szostak B.. (2022). The role of the human microbiome in the pathogenesis of pain. Int. J. Mol. Sci. 23(21), 13267. doi: 10.3390/ijms232113267, PMID: 36362056 PMC9659276

[B123] Valdés-Bango M. Gracia M. Rubio E. Vergara A. Casals-Pascual C. Ros C.. (2024). Comparative analysis of endometrial, vaginal, and gut microbiota in patients with and without adenomyosis. Acta Obstet. Gynecol. Scand. 103, 1271–1282. doi: 10.1111/aogs.14847, PMID: 38661227 PMC11168268

[B124] Vallvé-Juanico J. Houshdaran S. Giudice L. C. (2019). The endometrial immune environment of women with endometriosis. Hum. Reprod. Update. 25, 565–592. doi: 10.1093/humupd/dmz018, PMID: 31424502 PMC6737540

[B125] van Barneveld E. Manders J. van Osch F. H. M. van Poll M. Visser L. van Hanegem N.. (2022). Depression, anxiety, and correlating factors in endometriosis: A systematic review and meta-analysis. J. Women’s. Health 31, 219–230. doi: 10.1089/jwh.2021.0021, PMID: 34077695

[B126] Van Hul M. Cani P. D. Petitfils C. De Vos W. M. Tilg H. El-Omar E. M. (2024). What defines a healthy gut microbiome? Gut. 73(11), 1893–1908. doi: 10.1136/gutjnl-2024-333378, PMID: 39322314 PMC11503168

[B127] Velho R. V. Taube E. Sehouli J. Mechsner S. (2021). Neurogenic inflammation in the context of endometriosis—What do we know? Int. J. Mol. Sci. 22(23), 13102. doi: 10.3390/ijms222313102, PMID: 34884907 PMC8658724

[B128] Vetvicka V. Laganà A. S. Salmeri F. M. Triolo O. Palmara V. I. Vitale S. G.. (2016). Regulation of apoptotic pathways during endometriosis: from the molecular basis to the future perspectives. Arch. Gynecol. Obstet. 294, 897–904. doi: 10.1007/s00404-016-4195-6, PMID: 27628753

[B129] Vincent K. Warnaby C. Stagg C. J. Moore J. Kennedy S. Tracey I. (2011). Dysmenorrhoea is associated with central changes in otherwise healthy women. Pain 152, 1966–1975. doi: 10.1016/j.pain.2011.03.029, PMID: 21524851

[B130] Visconti A. Le Roy C.I. Rosa F. Rossi N. Martin T. C. Mohney R. P.. (2019). Interplay between the human gut microbiome and host metabolism. Nat. Commun. 10(1), 4505. doi: 10.1038/s41467-019-12476-z, PMID: 31582752 PMC6776654

[B131] Wang M.-Y. Sang L.-X. Sun S.-Y. (2024). Gut microbiota and female health. World J. Gastroenterol. 30, 1655–1662. doi: 10.3748/wjg.v30.i12.1655, PMID: 38617735 PMC11008377

[B132] Wang M. Zheng L. W. Ma S. Zhao D. H. Xu Y. (2024). The gut microbiota: emerging biomarkers and potential treatments for infertility-related diseases. Front. Cell. Infect. Microbiol. 14. doi: 10.3389/fcimb.2024.1450310, PMID: 39391885 PMC11464459

[B133] Wang X. Q. Zhou W. J. Luo X. Z. Tao Y. Li D. J. (2017). Synergistic effect of regulatory T cells and proinflammatory cytokines in angiogenesis in the endometriotic milieu. Hum. Reprod. 32, 1304–1317. doi: 10.1093/humrep/dex067, PMID: 28383711

[B134] Wang X. M M. Z. Song N. (2018). Inflammatory cytokines IL-6, IL-10, IL-13, TNF-α and peritoneal fluid flora were associated with infertility in patients with endometriosis. Eur. Rev. Med. Pharmacol. Sci. 22, 6. doi: 10.26355/eurrev_201805_14899, PMID: 29771400

[B135] Xiao F. Liu X. Guo S.-W. (2020). Platelets and regulatory T cells may induce a type 2 immunity that is conducive to the progression and fibrogenesis of endometriosis. Front. Immunol. 11. doi: 10.3389/fimmu.2020.610963, PMID: 33381124 PMC7767909

[B136] Xie S. Wang C. Song J. Zhang Y. Wang H. Chen X.. (2024). Lacticaseibacillus rhamnosus KY16 improves depression by promoting intestinal secretion of 5-HTP and altering the gut microbiota. J. Agric. Food Chem. 72, 14. doi: 10.1021/acs.jafc.4c03870, PMID: 39311539

[B137] Yang L. Ding W. Dong Y. Chen C. Zeng Y. Jiang Z.. (2022). Electroacupuncture attenuates surgical pain-induced delirium-like behavior in mice via remodeling gut microbiota and dendritic spine. Front. Immunol. 13. doi: 10.3389/fimmu.2022.955581, PMID: 36003380 PMC9393710

[B138] Young V. J. Ahmad S.F. Duncan W. C. Horne A. W. (2017). The role of TGF-β in the pathophysiology of peritoneal endometriosis. Hum. Reprod. Update. 23, 548–559. doi: 10.1093/humupd/dmx016, PMID: 28903471

[B139] Yu L. Shen H. Ren X. Wang A. Zhu S. Zheng Y.. (2021). Multi-omics analysis reveals the interaction between the complement system and the coagulation cascade in the development of endometriosis. Sci. Rep. 11(1), 11926. doi: 10.1038/s41598-021-90112-x, PMID: 34099740 PMC8185094

[B140] Yuan M. Li D. Zhang Z. Sun H. An M. Wang G. (2018). Endometriosis induces gut microbiota alterations in mice. Hum. Reprod. 33, 607–616. doi: 10.1093/humrep/dex372, PMID: 29462324

[B141] Zhong Q. Wu W. Xie J. Wang J.L. Xu K. Ren Y.. (2024) Limosilactobacillus-related 3-OMDP as a potential therapeutic target f or depression. Ann. Med. 56, 2417179. doi: 10.1080/07853890.2024.2417179, PMID: 39421970 PMC11492388

[B142] Zondervan K. T. Becker C. M. Koga K. Missmer S. A. Taylor R. N. Viganò P. (2018). Endometriosis. Nat. Rev. Dis. Primers 4 (1), 9. doi: 10.1038/s41572-018-0008-5, PMID: 30026507

